# Targeting the peripheral nervous system in adipose tissue with engineered AAV vectors

**DOI:** 10.1016/j.crmeth.2026.101542

**Published:** 2026-07-31

**Authors:** Jake W. Willows, Lindsey M. Lazor, Gilian Gunsch, Gargi Mishra, Andrew P. Villa, Timothy F. Shay, Andrew D. Steele, Kristy L. Townsend

**Affiliations:** 1 Department of Neurological Surgery, The Ohio State University, Columbus, OH, USA; 2 Biological Sciences Department, California State Polytechnic University Pomona, Pomona, CA, USA; 3 Division of Biology and Biological Engineering, California Institute of Technology, Pasadena, CA, USA

**Keywords:** adeno-associated virus, AAV, gene therapy, adipose, peripheral nervous system, DREADD, serotype, capsid

## Abstract

Adipose tissue innervation plays a critical role in regulating energy homeostasis and metabolic functions, yet targeted gene delivery to these peripheral nerves has remained challenging. We systematically evaluated both naturally occurring and engineered adeno-associated virus (AAV) capsids for their ability to transduce nerve fibers in the adipose tissue of mice. We compared seven AAVs (AAV1, AAVrg, AAV5, AAV9, AAV-PHP.S, AAV-MaCPNS1, and AAV-MaCPNS2) in C57BL/6J mice and identified AAV-PHP.S as highly efficient for transducing nerves within inguinal subcutaneous white adipose tissue (ing-scWAT). Titration studies further optimized intra-adipose delivery to minimize off-target expression while selectively targeting adipose tissue nerves. Building on this optimization, we employed Cre-dependent AAVs to selectively target Nav1.8^+^ sensory nerves in ing-scWAT, enabling Tetbow-based multicolor axon labeling and delivery of a chemogenetic effector. Together, these findings establish conditions for AAV transgene delivery to adipose nerves, providing opportunities for mechanistic studies and the development of therapies for neuropathic disorders.

## Highlights

• AAV-PHP.S enables efficient, selective gene delivery to adipose tissue nerves in mice
• We identify optimal AAV capsids and dosing to minimize off-target expression risks
• Cre-dependent AAVs target Nav1.8^+^ sensory nerves for multicolor axon mapping in fat
• The approach enables DREADD expression in adipose sensory nerves

## Motivation

Adipose tissue is densely innervated by peripheral nerves that regulate metabolic function and support bi-directional communication with the nervous system. However, tools for selectively labeling, mapping, and manipulating adipose-associated nerves remain limited. Existing AAV strategies have been optimized primarily for the CNS or other peripheral tissues, leaving adipose sensory and sympathetic circuits difficult to study. This work addresses that gap by defining AAV-based strategies to target sensory nerve fibers innervating subcutaneous adipose tissue. These optimized approaches support Tetbow-based multicolor axon labeling and chemogenetic activation of targeted sensory fibers, expanding experimental access to peripheral metabolic circuits.

## Graphical abstract

**Figure:**
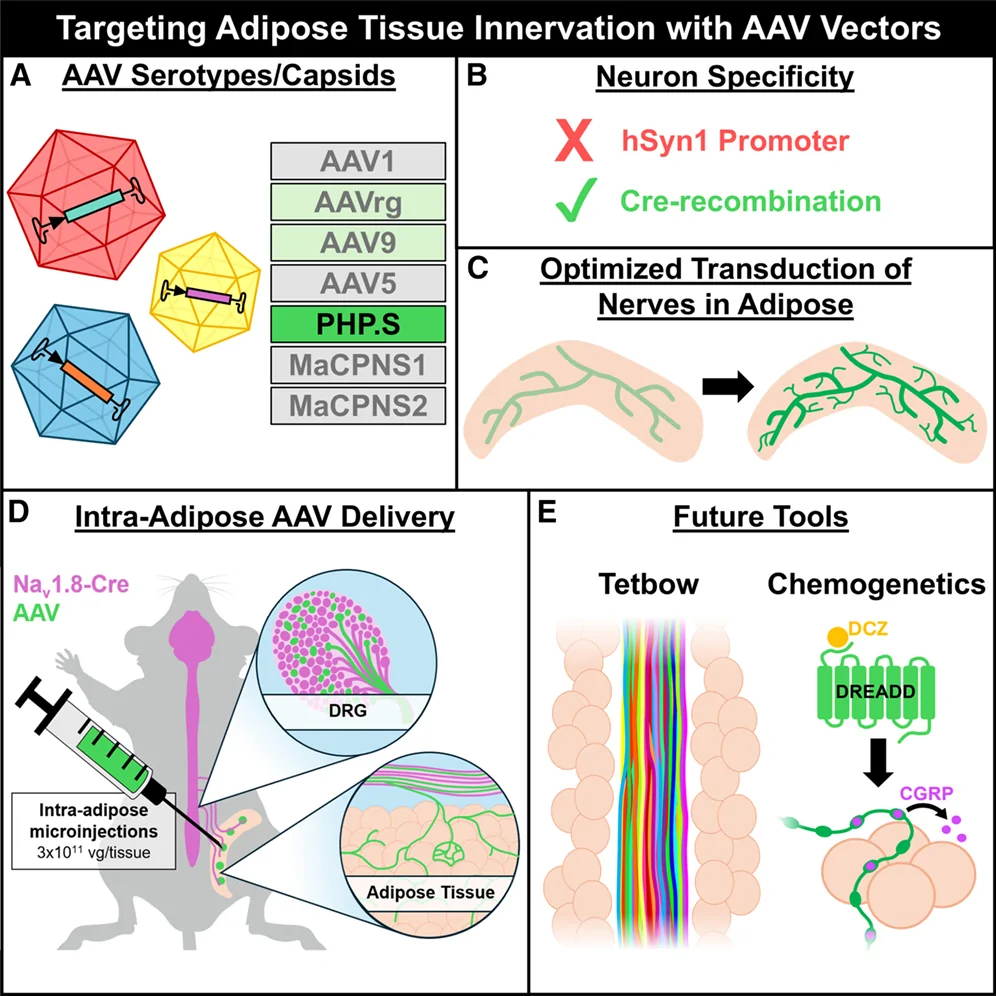

## Teaser

Peripheral innervation of adipose tissue is essential for metabolic regulation, yet gene delivery tools remain underdeveloped. Willows et al. establish a framework for targeting AAV-mediated transgene delivery to nerve subtypes in adipose tissue with Cre drivers, offering a promising tool for mechanistic studies and laying a framework for future therapeutic strategies targeting neuropathic disorders.

## Introduction

Recombinant adeno-associated viruses (rAAVs) are non-pathogenic parvoviruses composed of a single-stranded DNA genome of up to 4.7 kb, encapsulated within a non-enveloped icosahedral capsid. AAVs are routinely used as vectors to deliver transgenes to quiescent and slowly dividing cells, both as research tools and for clinical gene therapy applications.^1^ AAVs have been used extensively to study neurons in the central nervous system (CNS) and have become a staple tool for neuroscience research.^2^^,^^3^ Less work has been done to develop and optimize AAVs targeting the peripheral nervous system (PNS).^4^^,^^5^ Crucial for implementing AAVs in research is understanding the subtle differences among serotypes/capsids, in terms of tropism and directionality—whether the virus is taken up at the axon and transported toward the soma (retrograde transport) or taken up at the soma and transported to the axon terminal (anterograde transport).^3^ In general, AAVs are internalized by, firstly, the viral capsid adhering to the target cell via surface glycans or specific receptors.^6^ This is followed by the AAV being internalized into an endosome, from which it escapes to enter the nucleus. There, the viral genome becomes double stranded and is maintained largely as concatenated episomes.^6^ Uptake of AAVs into peripheral nerves is thought to occur primarily at distal nerve terminals, followed by retrograde transport to neuronal somata within sensory or autonomic ganglia, although direct uptake within the ganglia may also contribute, depending on how the AAV was administered.^7^^,^^8^ However, the molecular mechanisms underlying these processes remain poorly defined and appear to depend on capsid properties, delivery route, tissue context, and species.

There are thirteen naturally occurring AAV serotypes (AAV1–AAV13), each of which displays varying degrees of tropism for neurons and other cell types.^4^^,^^9^ Viral capsids can also be engineered to more effectively target specific cells and tissues, such as AAV-Rec2 (engineered to transduce adipocytes, as we have used previously^10^^,^^11^) and AAVrg (engineered to excel in retrograde transport^12^). While the AAV capsid ultimately dictates what cells can be transduced, they are typically associated with broad tropism across a mosaic of cell types and require cell-specific promoter sequences (such as the neuron-specific hSyn1 promoter) or Cre/Lox recombination to limit transgene expression to cells of interest.^3^ Currently, significant effort is being directed toward developing enhancer AAVs that incorporate neuronal subtype-specific regulatory elements upstream of the promoter. These regulatory sequences can greatly enhance cell type specificity and boost transgene expression in targeted neuronal populations.^13^^,^^14^^,^^15^^,^^16^ Currently, this approach has been developed exclusively to target neurons in the CNS.

Relatively little work has been done specifically to target peripheral nerves with AAVs; nonetheless, a few engineered AAVs (AAV-PHP.S, AAV-MaCPNS1, and AAV-MaCPNS2) have been shown to be efficient at transducing peripheral nerves when administered systemically via intravenous injection.^17^^,^^18^ All variants were engineered from the AAV9 capsid, chosen for its baseline affinity toward peripheral nerves, and were engineered with a 7-amino-acid insertion in a solvent-exposed variable region of each capsid. AAVs with peripheral transduction capabilities have proven invaluable for studying the enteric nervous system,^17^^,^^19^^,^^20^^,^^21^^,^^22^^,^^23^ cardiac ganglia,^24^ and dorsal root ganglia (DRGs),^22^^,^^25^ among several other tissues.^22^^,^^26^^,^^27^^,^^28^ Still, little effort has been placed into targeting the innervation of adipose tissue, a large and metabolically critical organ with an essential nerve supply.^29^^,^^30^^,^^31^^,^^32^

The hypothalamus, in conjunction with several extrahypothalamic brain regions, is responsible for the coordination and control of appetite and energy expenditure, in large part, through direct bi-directional neural communication with the body’s adipose tissue. Adipose tissue is densely innervated by efferent sympathetic and afferent sensory axons, which enables its bi-directional communication with the brain.^30^^,^^31^^,^^33^ Most adipose depots (primarily assessed in rodents), except for two minor brown adipose tissue (BAT) depots, lack parasympathetic innervation.^34^ The neural innervation of adipose tissue is required to regulate important metabolic processes, including lipolysis, adipogenesis, browning, non-shivering thermogenesis.^29^^,^^30^ Loss of adipose tissue innervation (“adipose neuropathy”) has been revealed in humans with aging or obesity,^35^ as well as in mouse models of aging^35^^,^^36^ and diabetic obesity,^35^^,^^37^^,^^38^^,^^39^ as we have previously demonstrated. Surgical and chemical denervation studies have demonstrated that the loss of healthy innervation in adipose negatively impacts whole-body metabolism and further exacerbates obesity-related pathologies.^30^ For this reason, it is crucial that efforts to develop therapies for peripheral neuropathies, including adipose neuropathy, be pursued. AAV-mediated tools will be critical for advancing adipose innervation research by enabling the same types of circuit-level approaches that have transformed hypothalamic studies, including optogenetic activation, chemogenetic modulation, and diphtheria toxin-mediated neuronal ablation.

For adipose tissue neuropathy, we have reported proof-of-concept gene therapy approaches by delivering neurotrophic factors via AAVs that transduced adipocytes, rather than manipulating the innervation itself.^37^ Work by the Bartolomucci and Zeltser Laboratories has shown that AAVs can be successfully leveraged to facilitate both optogenetic and chemogenetic neuromodulations of sympathetic nerves in BAT,^40^^,^^41^ indicating a therapeutic potential for treating obesity and diabetes. But, targeting specific neuronal subtypes in subcutaneous white adipose tissue (scWAT) has proven challenging. Common Cre-driver lines such as the “sympathetic” TH-Cre mice also express Cre-recombinase in a subset of sensory DRG neurons,^25^ as well as other cell types, making it ineffective at restricting transgene expression to only sympathetic neurons. AAV-PHP.S and AAV-MaCPNS1 driven by ubiquitous promoters (CAG and EF1α) and Cre-dependent DIO/flip-excision (FLEX) designs, have been injected into ganglia to anterogradely label inguinal scWAT (ing-scWAT) and perigonadal WAT (pgWAT) axons,^25^^,^^42^ and AAV-MaCPNS1 delivered into BAT has been shown to outperform AAVrg for the transduction of BAT-innervating sympathetic efferent fibers.^41^ Recently, the retrograde vector optimized for organ tracing (ROOT) was engineered from AAV9 to improve retrograde tracing from ing-scWAT to DRGs, which provides preferential tropism for DRG neurons but not sympathetic chain ganglia (SChG) neurons.^25^

In this study, we optimized adipose tissue sensory and sympathetic nerve transduction by AAVs through testing a variety of capsids and promoters, thereby developing a framework to assist researchers in incorporating peripheral AAVs into their studies, such as for manipulating nerve subtype activity through designer receptors exclusively activated by designer drugs (DREADDs). In addition to AAV9, PHP.S, MaCPNS1, and MaCPNS2, we also assessed AAV1, AAV5, and AAVrg. We systematically compared systemic and targeted intra-adipose (i.a.) AAV delivery approaches, evaluated commonly used promoter sequences (CAG, EF1α, and hSyn1), tested a range of viral titers, and examined Cre-dependent constructs to identify optimal strategies for selective transduction of peripheral nerves in adipose tissue. This approach yielded robust and reproducible transduction of adipose-associated peripheral nerves and enabled Tetbow multicolor labeling of adjacent axons in scWAT, as well as excitatory DREADD transgene expression in peptidergic axons within this depot. This work provides a comprehensive evaluation of AAV tropism in scWAT-projecting neurons, establishing a valuable resource for targeted neuromodulation and other mechanistic investigations in peripheral metabolic circuits.

## Results

### Determination of AAV capsids that effectively transduced adipose tissue nerves via systemic injection

Starting broadly, we assessed the transduction efficiencies of seven different AAV serotypes/capsids (AAV1, AAVrg, AAV5, AAV9, AAV-PHP.S, AAV-MaCPNS1, and AAV-MaCPNS2) when administered systemically (see Figure S1A for additional details on each). Male C57BL/6J mice received a single retro-orbital (r.o.) injection of 1 × 10^12^ vg/mouse, followed by a 4-week incubation period to provide sufficient time to achieve prominent transgene expression. Because systemic delivery typically requires the injected titer to be 1–2 orders of magnitude greater than that of a local injection,^4^ we started by injecting 1 × 10^12^ vg/mouse. Each AAV vector carried a fluorescent protein (FP) cargo (either tdTomato or EGFP) driven by the strong ubiquitous CAG promoter. A ubiquitous promoter sequence was used in the initial testing to achieve the most robust transduction. This offered no cell-type specificity other than that provided by the AAV capsid. Each tissue was compared with that from a saline-injected control mouse.

**Figure 1:**
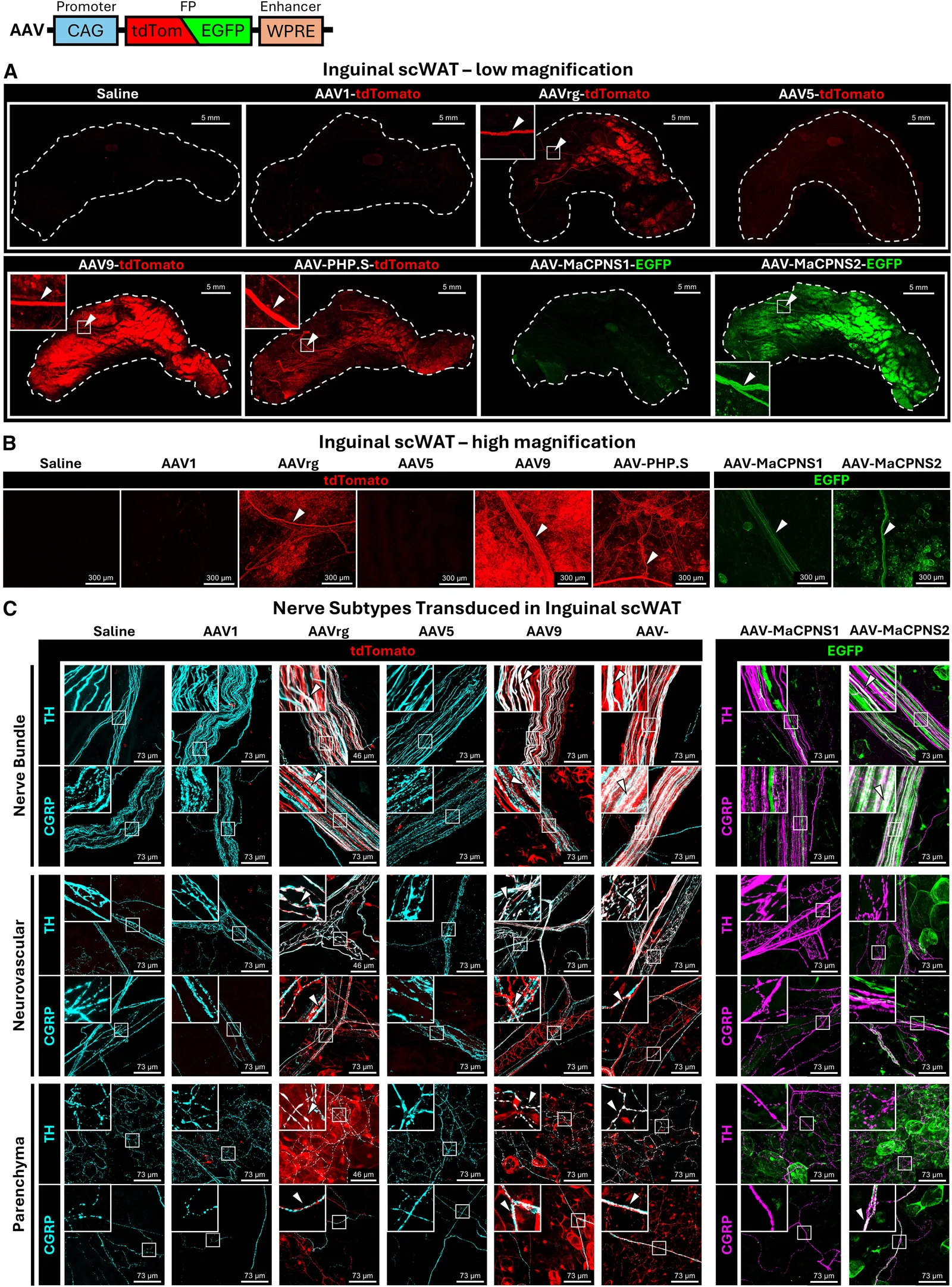
Identifying which AAV capsids effectively transduce adipose tissue nerves by systemic injection (A) Male and female C57BL/6J mice (16- to 19-week-old) were systemically injected (1 × 10^12^ vg/mouse, r.o.) with one of the seven AAV capsids (AAV1, AAVrg, AAV5, AAV9, PHP.S, MaCPNS1, or MaCPNS2), each carrying a fluorescent protein (FP) cargo—either tdTomato or EGFP—and compared against a saline-injected control mouse, AAV-CAG-tdTomato/EGFP-WPRE. Intact inguinal subcutaneous white adipose tissue (ing-scWAT) displayed as tiled *Z* maximum intensity (*Z*_max_) projections were captured at 10× objective magnification. Dashed lines indicate tissue boundaries; white boxes magnify the subiliac transverse nerves when visibly transduced, further identified with white arrows. (B) Representative 10× magnification *Z*_max_ projections of each ing-scWAT depot following AAV transduction. White arrows point to the transduced nerves. (C) High-magnification (40×–63×) representative micrographs of nerve bundles, vasculature, and tissue parenchyma in ing-scWAT co-labeled against either TH or CGRP. White boxes indicate magnified insets; white arrows point to examples of co-labeled (white) axons if present.

scWAT was excised and imaged to assess FP transgene expression across the intact depot (Figure 1A). Given that intracellular trafficking, capsid uncoating, and second-strand synthesis can decouple vector genome copies from functional expression, FP signal was used as the primary readout of productive transduction.^43^^,^^44^ The observed fluorescence intensity of neuronal structures was the primary determinant of AAV “success.” Bright FP expression not only indicated the extent of transduction but also eliminated the need to boost FP expression with antibodies, which would have added complexity and required additional controls to these experiments.

AAV1 and AAV5 displayed little to no transduction of ing-scWAT innervation, with only sparsely labeled adipocytes and immune cells. A coarse analysis of each whole ing-scWAT depot suggested that AAVrg, AAV9, PHP.S, MaCPNS2, and, to a lesser degree, MaCPNS1 all transduced adipose nerves, although the full extent was difficult to appreciate because adipocyte transduction obscured much of the tissue innervation. Most of the fluorescent signal observed in each tissue was from adipocytes (Figure 1A). The subiliac transverse nerves were easily identifiable at low magnification when successfully transduced (Figure 1A, white boxes). Higher-magnification imaging further confirmed the transduction of axons among other cells (Figure 1B).

Identifying a capsid capable of transducing multiple adipose-associated neural subpopulations was important because downstream specificity could then be achieved using defined Cre-driver lines. Therefore, we immunolabeled AAV-transduced tissues for tyrosine hydroxylase (TH), the rate-limiting enzyme in catecholamine synthesis and a marker commonly used to identify sympathetic fibers, and calcitonin gene-related peptide (CGRP), a neuropeptide expressed by peptidergic sensory neurons.^39^ TH has been used prolifically to label noradrenergic efferent axons in WAT and BAT,^45^^,^^46^^,^^47^^,^^48^^,^^49^ but it has also been shown now that a substantial percentage of the sensory afferent innervation of scWAT could be traced to TH^+^ cell bodies in the DRGs.^25^ Co-expression of the FP transgene with both TH^+^ and CGRP^+^ axons was observed by AAVrg, AAV9, and PHP.S within nerve bundles, around vasculature, and within the tissue parenchyma (Figure 1C). MaCPNS1 failed to label axons outside the large nerve bundles, and MaCPNS2 failed to label TH^+^ axons, while demonstrating a strong preference for CGRP^+^ axons (Figure 1C).

While our focus was on adipose innervation, we also assessed other relevant tissues from the same mice, including the DRGs at thoracic level 12 (given that T11-L3 DRGs innervate ing-scWAT^31^), the sciatic nerve, neuromuscular junctions (NMJs) in the soleus muscle, and the liver (Figure S1B). Briefly, PHP.S and MaCPNS2 labeled the most cell bodies in the DRGs and displayed the brightest FP expression in the sciatic nerve. AAVrg displayed the least expression in the liver, and none of the AAVs were observed to transduce NMJs of the soleus muscle, potentially because these are motor axons.

In our initial screening, AAVrg, AAV9, and AAV-PHP.S exhibited the highest potential for effective adipose tissue nerve transduction. To validate these findings, we conducted a replicate experiment focusing on these three vectors (Figures S1C–S1E). Of note, all three capsids were confirmed to transduce the neuro-adipose nexus (NAN; specialized axon terminal structures in scWAT that we discovered previously^30^^,^^39^^,^^50^) (Figure S1F). PHP.S outperformed AAVrg and AAV9 within the DRGs (Figure S1E).

In the originating study, systemic delivery of PHP.S achieved transduction in 82% of the assessed DRG neurons. It demonstrated several advantages over AAV9, including enhanced transduction of sensory afferents, DRGs, cardiac ganglia, and the myenteric plexus.^18^ A subsequent study reported that PHP.S transduced the rat DRGs and pelvic ganglia similarly to AAV9 and AAVrg; however, PHP.S demonstrated preferential tropism for nNOS^+^ parasympathetic neurons in the pelvic ganglia, whereas AAVrg showed enhanced tropism for sympathetic neurons in the pelvic ganglia. In the DRGs, both PHP.S and AAVrg effectively transduced large-diameter myelinated neurons (NF200^+^), small-diameter peptidergic neurons (CGRP^+^), and non-peptidergic neurons (IB4^+^) at comparable rates. Notably, PHP.S slightly preferred NF200^+^ and CGRP^+^ neurons compared with AAVrg. Despite these observations, PHP.S did not significantly outperform AAV9 in rats.^22^ Surprisingly, when AAV was directly injected into the DRGs, PHP.S was outperformed by AAVrg, AAV8, and AAV9, all of which exhibited comparable expression levels.^51^

Together, these data identified AAVrg, AAV9, and AAV-PHP.S as the most promising capsids for adipose nerve transduction. Due to the enhanced labeling in the DRGs and sciatic nerves, we decided to proceed with PHP.S. Figure S1G summarizes relative qualitative comparisons for each AAV capsid and the extent of transduction of each tissue assessed.

### Optimization of systemic and intra-adipose AAV delivery

While the CAG promoter exhibited robust transgene expression in scWAT, we also tested two additional widely used promoter sequences: EF1α, an alternative strong ubiquitous promoter sequence smaller than CAG, and hSyn1, a commonly used neuron-specific promoter. C57BL/6J mice received systemic delivery of AAV9-EF1α-EGFP or AAV9-hSyn1-EGFP at a titer of 1 × 10^12^ vg/mouse (r.o.). Ing-scWAT was excised and evaluated for EGFP expression. The EF1α promoter noticeably blunted transduction of non-neuronal cells in scWAT (adipocytes, immune cells, and axons) compared with CAG but still transduced TH^+^ and CGRP^+^ axons throughout the tissue (Figure S2A). As expected, the hSyn1 promoter further reduced adipocyte and immune cell transduction but not entirely (Figure S2B). Unexpectedly, hSyn1 restricted axonal transgene expression in scWAT to only a few TH^−^/CGRP^−^ double-negative axons (thereby presumed to be non-peptidergic sensory axons), and the expression was not improved by boosting GFP fluorescence with immunolabeling. While this was unexpected, as the large majority of ing-scWAT peripheral innervation expresses SYN1 (Figure S2C), it is consistent with previous studies that reported reduced peripheral nerve transduction upon replacing ubiquitous promoters with hSyn1.^19^^,^^52^ Specifically, relative to CAG-driven constructs, hSyn1-driven expression substantially diminished the labeling of DRGs and jugular-nodose neurons and did not yield detectable labeling of axonal projections in the vagus nerve or dorsal roots.^19^ Based on these observations, we concluded that sufficient neuron-specific transgene expression in scWAT would require a strong ubiquitous promoter while using Cre-mediated recombination to restrict the expression to axon subtypes, as the neuron-specific promoter alone proved too restrictive.

To reduce the viral titer required for each systemic injection (1 × 10^12^ vg/mouse), we performed a titration of 5 × 10^11^, 3 × 10^11^, and 1 × 10^11^ vg/mouse of the AAV-PHP.S-CAG-tdTomato systemically delivered to male and female C57BL/6J mice. For each, we assessed the ing-scWAT depot, DRGs, and sciatic nerve to determine the transduction efficiency. Our findings revealed that 5 × 10^11^ vg/mouse produced FP transgene expression levels comparable to those observed with 1 × 10^12^ vg/mouse. Reducing the dose to 3 × 10^11^ vg/mouse resulted in a pronounced decline in FP expression across all assessed tissues (Figure S2D). Investigating further, we immunolabeled T12 DRG sections with PGP9.5 (pan-neuronal marker) with either TH or CGRP to assess if the viral titer impacted co-expression of the transgene with relevant neural subtypes. Even though lower viral titers appeared to reduce the number of cells transduced in the DRGs, similar co-expression with TH^+^ or CGRP^+^ cell bodies was observed at all titers (Figure S2E).

**Figure 2:**
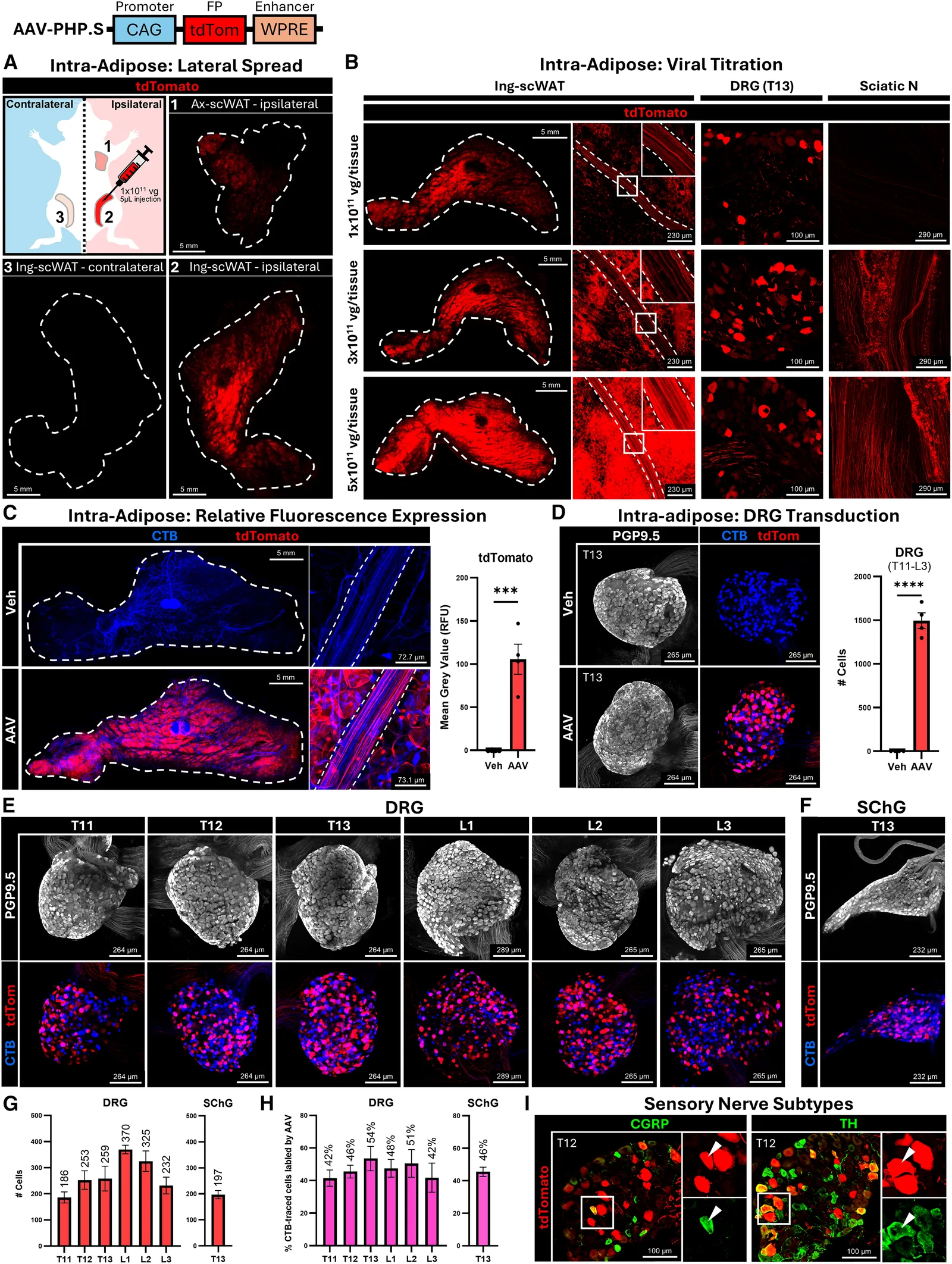
AAV-PHP.S for targeted intra-adipose delivery (A) Intra-adipose (i.a.) transdermal microinjection of AAV-PHP.S-CAG-tdTomato-WPRE (1 × 10^11^ vg/tissue, i.a.) into the ipsilateral ing-scWAT of a male C57BL/6J mouse (17-week-old). Non-injected axillary (ax)-scWAT from the ipsilateral side and ing-scWAT from the contralateral side were excised intact and imaged to assess the lateral spread of AAV. Tissue boundaries are indicated with white dashed lines. (B) Male and female C57BL/6J mice (12- to 17-weeks old) received AAV-PHP.S-CAG-tdTomato-WPRE by i.a. injection. Fluorescent transgene expression was compared across three viral titers for i.a. delivery: 1 × 10^11^, 3 × 10^11^, and 5 × 10^11^ vg/tissue. Tissues assessed included ing-scWAT with a representative image of nerve bundle (between parallel dashed lines, magnified in white box), T13 DRG, and sciatic nerve. (C) Female C57BL/6J mice (*n* = 4, 10- to 19-weeks old) received AAV-PHP.S-CAG-tdTomato-WPRE (red) or saline vehicle by i.a. injection. Four days prior to tissue excision, all mice received CTB-647 (blue) by i.a. injection to retrogradely trace scWAT-innervating neurons to the ganglia. Representative images of ing-scWAT with an example of a high-magnification nerve bundle (between parallel dashed lines). Relative tdTomato transgene expression was quantified by the mean fluorescence intensity. ∗∗∗*p* < 0.001, by Student’s *t* test. Error bars are ± SEM. (D) Representative images of whole T13 DRGs immunolabeled against PGP9.5 (cell bodies in gray) comparing AAV- and vehicle-injected mice. Quantification of transduced DRG cells (combined levels T11-L3) (*n* = 4). ∗∗∗∗*p* < 0.0001, by Student’s *t* test. Error bars are ± SEM. (E) Representative whole T11-L3 DRGs following AAV (red) and CTB (blue) i.a. delivery. Co-labeled cells are shown in magenta. PGP9.5 is used to show the ganglion structure (gray). (F) Representative whole T13 SChGs following AAV (red) and CTB (blue) i.a. delivery. Co-labeled cells are shown in magenta. PGP9.5 is used to show the ganglion structure (gray). (G) Number of cells transduced per level of ganglia (*n* = 4). Error bars are ± SEM. (H) Percentage of CTB-traced cells transduced by AAV at each level of ganglia (*n* = 4). Error bars are ± SEM. (I) Thin-sectioned T12 DRG immunostained against either TH (green) or CGRP (green) to assess the impact of viral titer on co-expression. White boxes indicate zoomed regions, and white arrows point to the examples of co-labeled cells (yellow).

While systemic AAV delivery demonstrated broad applicability in transducing adipose tissue among other tissues simultaneously, targeting a specific adipose depot would be preferable for most experiments, such as neuromodulatory studies, to manipulate a depot’s distinct nerve activity. To address this need, we tested the feasibility of i.a. transdermal micro-injections by injecting 5 μL of 1 × 10^11^ vg into the ing-scWAT depot of a male C57BL/6J mouse, followed by a 4-week incubation. We identified FP expression in the injected ing-scWAT depot and, to a lesser extent, within the adjacent axillary (ax-)scWAT depot (Figure 2A), which shares a lymphatic supply with ing-scWAT. Transduced cells were absent from the contralateral ing-scWAT depot (Figure 2A).

A similar viral titration was performed for i.a. transdermal microinjections (5 × 10^11^, 3 × 10^11^, and 1 × 10^11^ vg/tissue) with AAV-PHP.S-CAG-tdTomato injected unilaterally into ing-scWAT of C57BL/6J mice. Higher viral titers, such as 1 × 10^12^ vg, were also tested, but visible tdTomato expression in the surrounding tissues (e.g., peritoneum) at the time of dissection indicated substantial off-target transduction, and these titers were, therefore, excluded from further analysis. All titers effectively transduced DRG neurons innervating ing-scWAT (Figure 2B). 3 × 10^11^ vg/tissue (∼15 μL) was sufficient to fully saturate the tissue (equal fluorescence intensity across the depot) but with noticeable spillover into surrounding tissues, as indicated by FP expression in the sciatic nerve (Figure 2B). Injecting 1 × 10^11^ vg/tissue (∼5 μL) had heterogeneous labeling in ing-scWAT, with FP expression slightly more abundant around the injection site, but the lower volume eliminated spillover to the adjacent tissues (Figure 2B).

Female C57BL/6J mice received microinjection of AAV-PHP.S-CAG-tdTomato (1 × 10^11^ vg/tissue, i.a., 15 μL) (*n* = 4) or an equal volume of saline vehicle (*n* = 4) to quantitatively assess tdTomato transgene expression across ing-scWAT, DRGs, and the SChGs. After a four-week viral incubation, i.a. microinjection of fluorescently labeled cholera toxin subunit B (CTB) retrogradely labeled ing-scWAT-innervating axons to their soma in the DRGs and SChGs. CTB incubated for 4 days prior to tissue collection.

CTB broadly labeled most cells in ing-scWAT, and the tdTomato transgene was only detectable in the AAV-injected depot (*p* = 0.0009; Figure 2C). Similarly, CTB effectively traced to the adipose-innervating ganglia and only in mice that received AAV expressed tdTomato labeling (*p* < 0.0001; Figure 2D). tdTomato expression was detected in the assessed DRGs (T11-L3) (Figure 2E) and SChGs (T13) (Figure 2F), with the greatest number of cells transduced at DRG L1 (Figure 2C). On average, approximately 42%–54% of the CTB-traced neurons were transduced (Figure 2H). As with systemic injection, both TH^+^ and CGRP^+^ cells in the DRGs expressed the transgene (Figure 2I).

Systemic administration enables broad neural transduction of the adipose tissue, while i.a. injection offers precise, depot-specific targeting for neuromodulation. Here, we have optimized both approaches to provide researchers with flexible strategies to align with their experimental goals.

### AAV-PHP.S Cre-mediated transgene expression in ing-scWAT

FLEX vectors are engineered so that two pairs of Lox sites flank the transgene, and its open reading frame is inverted in relation to the promoter. This allows for Cre-recombinase-mediated transgene expression. To test if the systemic transgene delivery using a Cre-mediated approach was sufficient for targeting the innervation of adipose tissue, we injected AAV-PHP.S-CAG-FLEX-tdTomato systemically (1 × 10^12^ vg/mouse, r.o.) into an adult male Na_v_1.8-Cre::ZsGreen1 mouse (sensory nerve reporter). Unexpectedly, this titer failed to transduce most sensory innervation in ing-scWAT, apart from the large-diameter nerve bundles (Figure S3A). These results suggested that achieving robust Cre-mediated transgene expression in the adipose parenchyma required substantially higher viral titers. For example, i.a. microinjection of 1 × 10^11^ vg/tissue produced limited transduction of small fibers in ing-scWAT, with heterogeneous expression across the tissue and most pronounced expression near the injection site (Figure S3B). Increasing the injection titer by 3-fold (to 3 × 10^11^ vg/tissue i.a., or 3 × 10^12^ vg/mouse, r.o.) markedly enhanced the transduction efficiency.

With this in mind, we injected female Na_v_1.8-Cre::ZsGreen1 (Cre^+/−^, *n* = 4) and ZsGreen1 mice (Cre^−/−^, *n* = 4) with AAV-PHP.S-CAG-FLEX-tdTomato (3 × 10^11^ vg/tissue, i.a.) to assess the transduction efficiency of ing-scWAT axons, using a Cre-dependent approach. Additionally, Cre^+/−^ mice received equal-volume microinjections of saline vehicle into their contralateral depot as an additional control.

**Figure 3:**
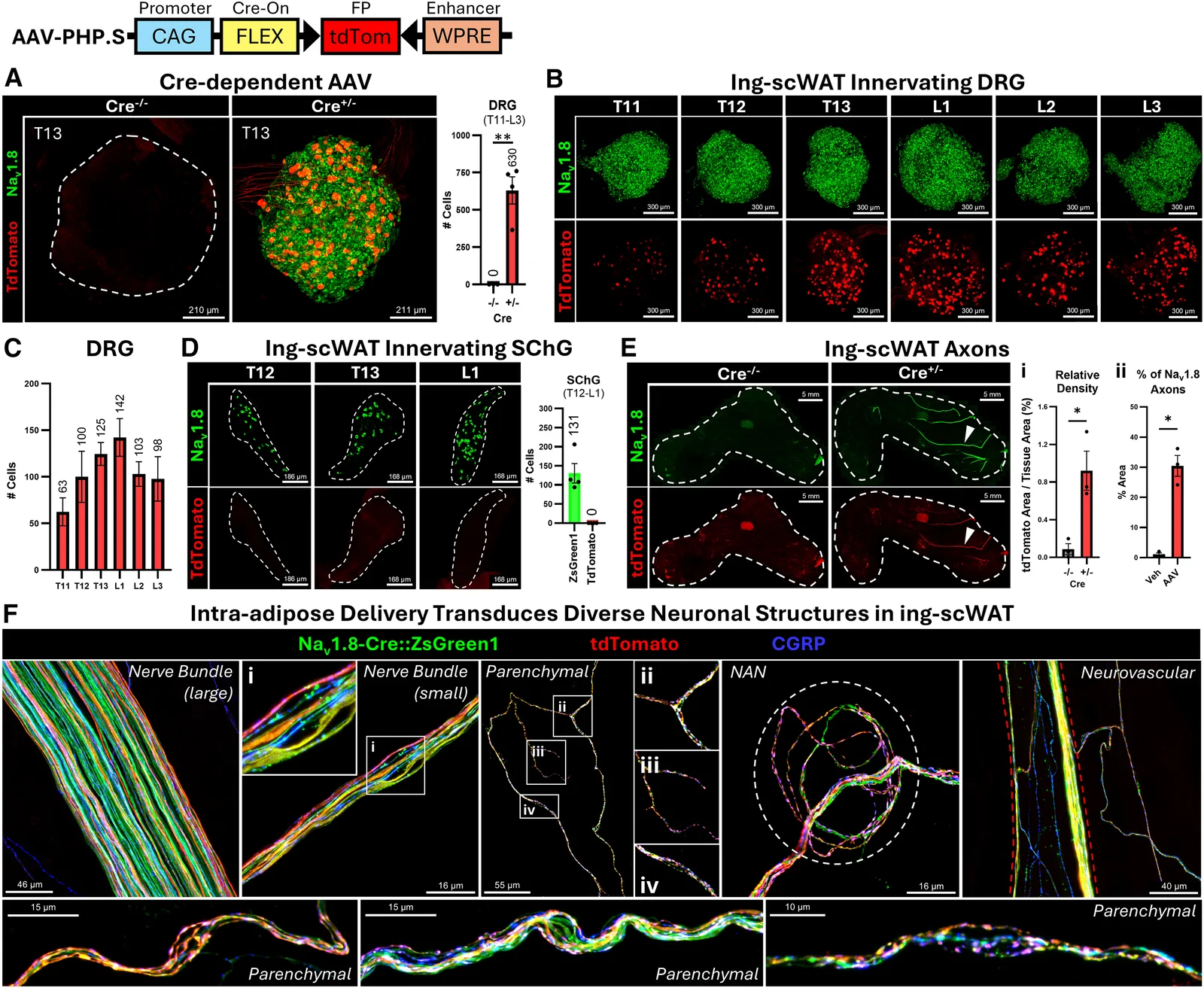
Cre-mediated AAV-PHP.S transduction of adipose tissue sensory nerves by intra-adipose injection (A) Female Na_v_1.8-Cre::ZsGreen1 (Cre^+/−^) and ZsGreen1 (Cre^−/−^) mice (*n* = 3–4, 19-23-week-old) received AAV-PHP.S-CAG-FLEX-tdTomato-WPRE (3 × 10^11^ vg/tissue, i.a.). Cre^+/−^ mice also received an injection of saline-vehicle into their contralateral ing-scWAT depot. Representative images of whole T13 DRGs (ZsGreen1 in green; AAV-tdTomato in red), with quantification of transduced DRG cells (combined levels T11-L3) (*n* = 3–4). Ganglia from Cre^−/−^ mouse is outlined in white dashes. ∗∗*p* < 0.01, by Student’s *t* test. Error bars are ± SEM. (B) Representative images of whole T11-L3 DRGs of Cre^+/−^ mice (Na_v_1.8^+^, green; AAV-tdTomato, red). (C) Number of cells transduced per level of DRGs (*n* = 4). Error bars are ± SEM. (D) Representative images of whole T12-L1 SChGs of Cre^+/−^ mice. Ganglion boundaries are outlined with white dashes. Number of Na_v_1.8^+^ cells (green) and AAV transduced cells (red) (n = 4). Error bars are ± SEM. (E) Representative images of intact ing-scWAT. (i) Relative nerve density of TdTomato^+^ axons compared between Cre^+/−^ and Cre^−/−^ mice (*n* = 3). (ii) Percent area of Na_v_1.8^+^ axons co-labeled by tdTomato, compared between AAV-injected and vehicle-injected contralateral depots of Cre^+/−^ mice (*n* = 3). ∗*p* < 0.05, by Student’s *t* test. Error bars are ± SEM. (F) AAV-PHP.S-CAG-FLEX-tdTomato-WPRE was injected (3 × 10^11^ vg/tissue, i.a.) into a male Na_v_1.8-Cre::ZsGreen1 mouse (21-week-old). Representations of transduced nerve bundles, vascular innervation (red dashed lines), parenchymal innervation, and NANs (white dashed circle) are shown. Na_v_1.8^+^ axons (green), CGRP^+^ axons (blue), and AAV-transduced axons (red). White boxes show magnified insets (i–iv).

In the DRGs, tdTomato transgene was expressed only in Cre^+/−^ mice (*p* = 0.0021; Figure 3A). tdTomato-labeled neurons were detected in T11–L3 DRGs (Figure 3B), with peak transduction at L1 and progressively fewer labeled neurons in the adjacent rostral and caudal ganglia (Figure 3C). This pattern is similar to that observed with the Cre-independent approach (Figure 2G), as should be expected based on previous studies that mapped the ing-scWAT neuronal input.^31^^,^^53^ The Cre-dependent AAV approach transduced fewer cells in the DRGs, presumably due to the greater specificity of the Cre-driver compared with that provided by the ubiquitous promoter.

Although Na_v_1.8 is typically a marker of sensory afferents, some reports have identified a subset of Na_v_1.8^+^ sympathetic neurons,^54^^,^^55^ consistent with our current observation of an average of 131 Na_v_1.8^+^ neurons within the combined T12-L1 SChGs (Figure 3D). Despite this, tdTomato transgene expression was not observed in any SChG neuron (Figure 3D), presumably because they do not innervate ing-scWAT. Within ing-scWAT, tdTomato expression was prominently visible in large Na_v_1.8^+^ nerve bundles and only in Cre^+/−^ mice (*p* = 0.0182; Figure 3E). tdTomato labeling that overlapped with ZsGreen1 was quantified relative to the contralateral vehicle-injected depot. Approximately 30% of the Na_v_1.8^+^ axonal area co-expressed tdTomato (*p* = 0.0108; Figure 3E), which we suspect is a conservatively low estimate due to much of the tdTomato labeling failing to substantially overcome autofluorescence for this analysis. tdTomato expression was almost entirely confined to Na_v_1.8^+^ axons, many of which co-expressed CGRP. High-magnification representative images illustrate co-labeled axons within both large- and small-diameter nerve bundles, around vasculature, and axons throughout the adipose parenchyma, and forming NANs (Figure 3F). A small subset of adipocytes expressed the tdTomato transgene due to off-target Cre recombination (Figure S3C). This off-target expression is common with Cre-dependent AAVs, even in wild-type mice,^56^ and highlights the importance of thoroughly assessing and documenting the distribution of expression for each experiment or combination of tissue/virus/Cre-line.

### Testing the broader applicability of Cre-dependent AAV-PHP.S in adipose tissue

**Figure 4:**
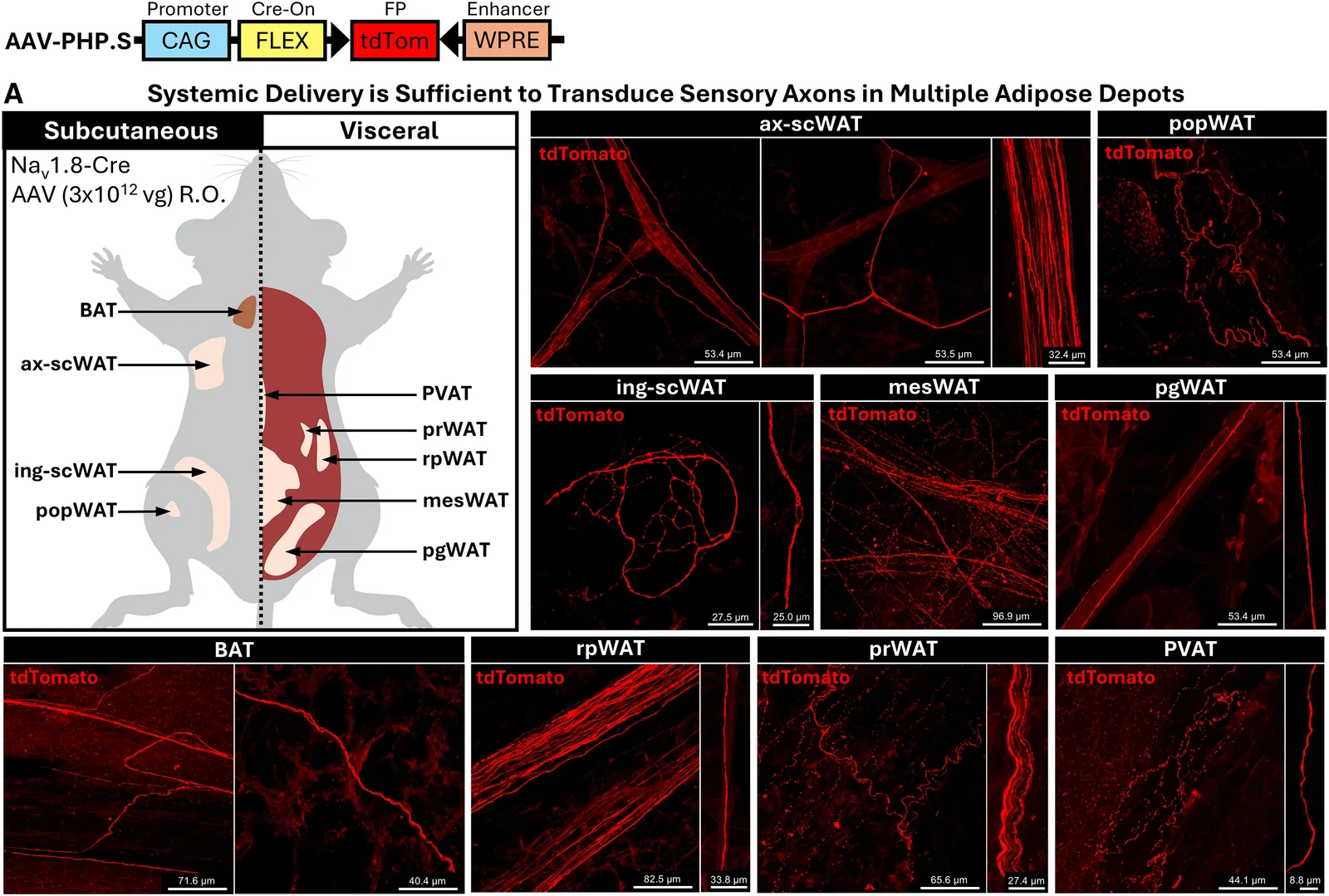
Systemic delivery of Cre-dependent AAV transduced sensory innervation of multiple adipose depots (A) AAV-PHP.S-CAG-FLEX-tdTomato-WPRE was injected systemically (1 × 10^12^ vg/mouse, r.o.) into a male Na_v_1.8-Cre mouse (21-week-old). Multiple adipose depots were excised intact and whole-mount processed for imaging. Adipose depots assessed included: BAT, axillary scWAT (ax-scWAT), ing-scWAT, popliteal WAT (popWAT), mesenteric WAT (mesWAT), perigonadal WAT (pgWAT), retroperitoneal WAT (rpWAT), perirenal WAT (prWAT), and perivascular adipose tissue (PVAT) from the abdominal aorta. Transduced axons (tdTomato, red).

To assess whether this approach may be sufficient for adipose depots other than scWAT, we systemically injected AAV-PHP.S-CAG-FLEX-tdTomato (3 × 10^12^ vg/mouse, r.o.) into a male Na_v_1.8-Cre mouse and excised several adipose depots across the body: axillary scWAT (ax-scWAT), ing-scWAT, popliteal WAT (popWAT), mesenteric WAT (mesWAT), pgWAT, retroperitoneal WAT (rpWAT), perirenal WAT (prWAT), interscapular BAT, and perivascular adipose tissue (PVAT) from the abdominal aorta. We observed tdTomato transgene expression within axons across all assessed adipose depots (Figure 4A).

**Figure 5:**
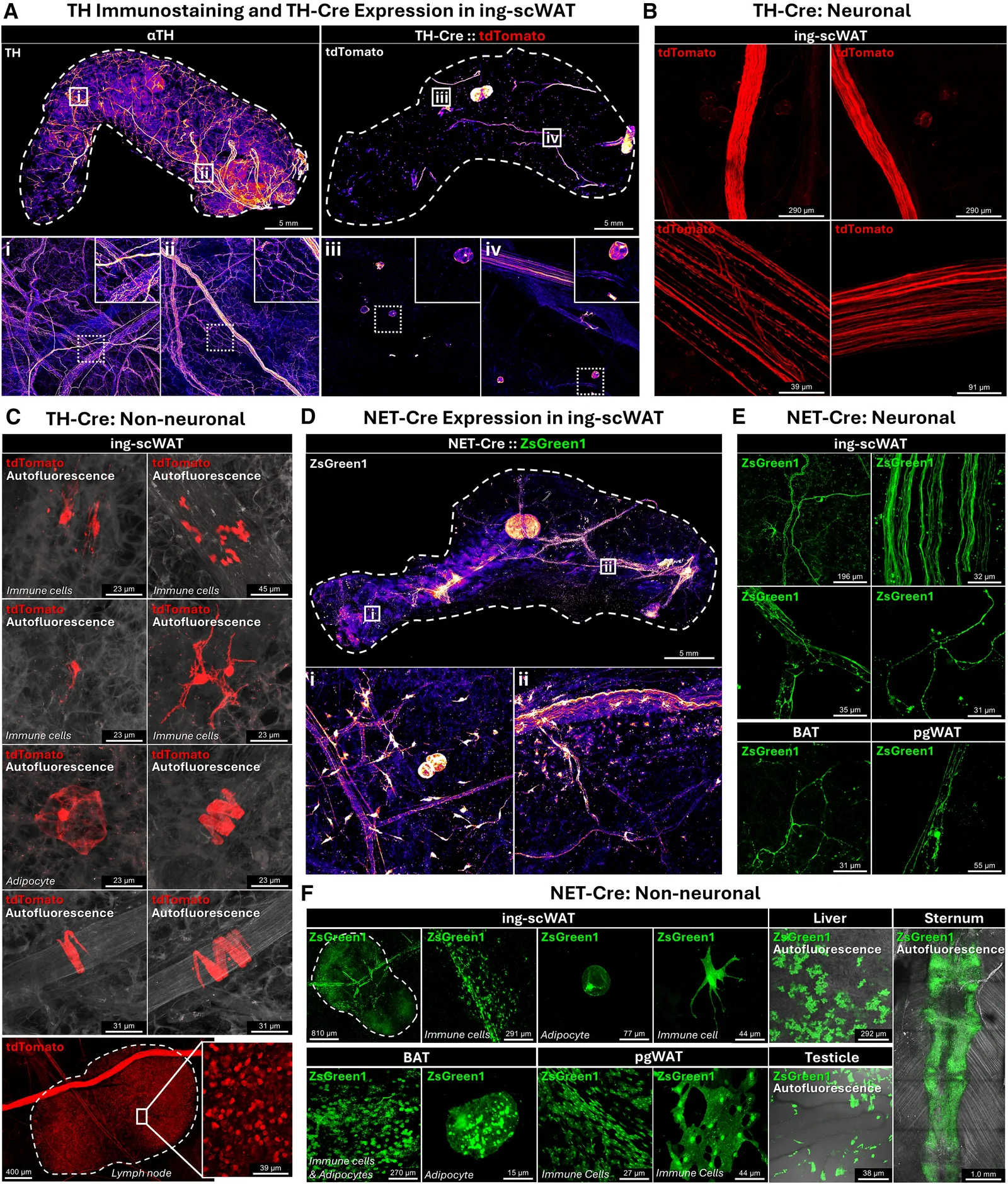
Characterization of TH-Cre and NET-Cre recombination in adipose and other peripheral tissues (A) Intact ing-scWAT depot was excised either from a male C57BL/6J mouse (12-week-old) and immunostained against tyrosine hydroxylase (αTH), or from a male TH-Cre::tdTomato mouse (12-week-old). Fluorescence intensity (LUT: Fire). Representative images from each tissue (white solid boxes, i–iv), with regions (white dotted boxes) further magnified. (B) Representative images of TH-Cre expression in axons within ing-scWAT of TH-Cret::Tomato mice (red). Labeled axons are primarily restricted to those contained in large-diameter nerve bundles. (C) Representative images of TH-Cre expression (red) in non-neuronal cells (immune cells and adipocytes) within ing-scWAT of TH-Cre::tdTomato mice. (D) Intact ing-scWAT depot excised from male and female norepinephrine transporter (NET)-Cre::ZsGreen1 mice (20-week-old) (LUT: Fire). White boxes (i and ii) show magnified regions. (E) Representative images of NET-Cre expression (green) in axons within ing-scWAT, BAT, and pgWAT. (F) Representative images of NET-Cre expression (green) in non-neuronal cells within ing-scWAT, BAT, pgWAT, liver, testes, and sternum. White dashes outline the subiliac lymph node.

We also sought to demonstrate Cre-mediated transgene expression in TH-Cre mice. Unfortunately, we found that the available TH-Cre lines were unreliable in ing-scWAT due to weak neuronal fluorescence and leaky Cre expression in many cell types. A stark contrast was observed when comparing TH immunolabeling against TH-Cre::tdTomato mice (Figure 5A). The large-diameter nerve bundles in ing-scWAT expressed tdTomato, but the majority of innervation (including neurovascular innervation and small varicose axons in the parenchyma) did not (Figure 5B). Instead, off-target Cre-recombinase activity was observed in the subiliac lymph node and several other cell types, including adipocytes and immune cells (Figure 5C). Similar results were observed in the ing-scWAT from TH-Cre mice that were obtained from the EMMA repository or when crossed with alternative fluorescent reporters, ZsGreen1 and EYFP (data not shown). This is particularly concerning because several influential studies have used these mice to investigate adipose tissue innervation.^40^^,^^45^^,^^47^ Moreover, emerging evidence that TH^+^ sensory nerves innervate ing-scWAT underscores the need to pursue mice with Cre expression specific to sympathetic nerves.^25^

To identify an alternative to TH-Cre for targeting sympathetic nerves, we assessed norepinephrine transporter (NET)-Cre mice. Ing-scWAT excised from NET-Cre::ZsGreen1 mice demonstrated prominent fluorescence in nerves across the tissue, but this was largely overshadowed by the various other cell types that express NET, which were visually much brighter than the axons (Figure 5D). NET-Cre recombination was observed in the axons innervating ing-scWAT, BAT, and pgWAT (Figure 5E). NET-Cre expression was also observed in non-neuronal cells in ing-scWAT, BAT, pgWAT, the liver, testes, and sternum (Figure 5F). NET-Cre expression was relegated to the cells known or suspected to express NET (immune cells and brown and beige adipocytes), unlike TH-Cre expression, which seemed largely off-target (Figures 5B and 5C). We ultimately decided not to perform AAV experiments with TH-Cre or NET-Cre mice due to the extensive non-neuronal expression observed in each mouse line.

While AAV-PHP.S-FLEX enables Cre-dependent transgene expression across adipose, the effectiveness is constrained by the availability and quality of applicable Cre-driver lines. Furthermore, unexpected recombination events, whether thoroughly assessed by researchers or not, and whether appropriate controls were included, are often under-reported in the literature. For this reason, Cre-lines should be thoroughly evaluated for off-target and leaky/ectopic Cre expression before experimentation, regardless of what has been previously reported in the literature and should be additionally assessed in the tissue of interest with the AAV transduction. For more information on designing Cre-lox experiments, we recommend a review by Song and Palmiter.^57^

### Tetbow multicolor labeling of adjacent axons in ing-scWAT

**Figure 6:**
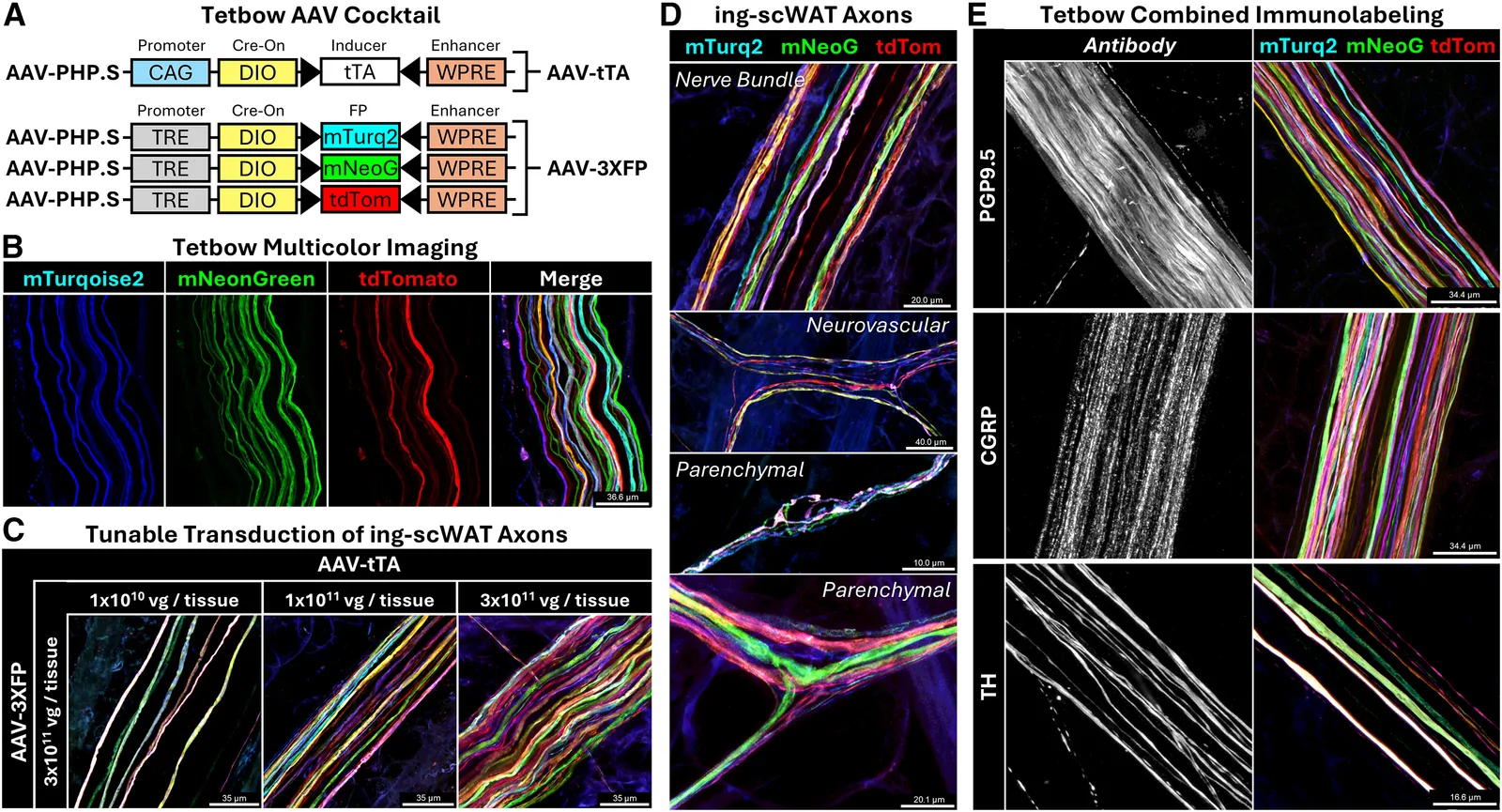
Tetbow multicolor labeling of ing-scWAT axons (A) Schematic of Tetbow AAV Cocktail used for intra-adipose transdermal microinjection into ing-scWAT. (B) Male Na_v_1.8-Cre mouse (9-week-old) received Tetbow AAV cocktail (AAV-tTA, 1 × 10^11^ vg/tissue; AAV-3XFP, 3 × 10^11^ vg/tissue/XFP; i.a.). Color separation of mTurquoise, mNeonGreen, and tdTomato within ing-scWAT nerve bundle. (C) Comparison of AAV-tTA viral titers (1 × 10^10^, 1 × 10^11^, and 3 × 10^11^ vg/tissue, i.a.) to induce transgene expression. All mice received 3 × 10^11^ vg/tissue (i.a.) of each of the three XFP vectors. (D) Examples of multicolor labeling of adjacent axons in ing-scWAT. (E) Tetbow combined with immunolabeling (gray) against either PGP9.5, CGRP, or TH, demonstrating the expanded capabilities of this tool in ing-scWAT.

Our next goal was to demonstrate how these viral tools could be applied within adipose tissue to enable new lines of investigation in adipose neurobiology. Using our optimized Cre-dependent strategy in combination with Tetbow,^18^ we achieved multicolor labeling of adjacent sensory axons in ing-scWAT by injecting Na_v_1.8-Cre mice with a cocktail of four compatible AAVs (Figure 6A). The first component of the cocktail was AAV-PHP.S-CAG-DIO-tTA (“AAV-tTA”), in which tTA is driven by a ubiquitous CAG promoter but expresses only following Cre-mediated recombination of the DIO/FLEX cassette. tTA then serves as the transcriptional activator for the remaining three AAVs, each encoding a spectrally distinct FP, AAV-PHP.S-TRE-DIO-XFP (“AAV-3XFP”). Activation of the Tet-responsive element (TRE) by tTA resulted in stochastic expression of mTurquoise2, mNeonGreen, and tdTomato, producing a mosaic of hues (Figure 6B). Using this two-component system, the brightness and diversity of multicolor expression are uncoupled from the axon labeling density,^18^^,^^58^ or in other words, by holding the AAV-3XFP titer constant (3 × 10^11^ vg/tissue/XFP) and varying the AAV-tTA titer (1 × 10^10^, 1 × 10^11^, and 3 × 10^11^ vg/tissue), we selectively controlled the number of adjacent axons labeled without affecting the fluorescence intensity or color diversity (Figure 6C). This allowed for the differentiation of adjacent sensory axons in ing-scWAT, with examples provided in Figure 6D. This tool was expanded further by combining Tetbow with immunofluorescence labeling (Figure 6E). In our hands, for labeling peripheral nerves in adipose, Tetbow proved markedly superior to traditional Brainbow approaches.^59^^,^^60^^,^^61^ Brainbow3.2 worked well in differentiating adjacent axons within the sciatic and optic nerves (Figures S4A and S4B) but failed to consistently label ing-scWAT nerve bundles with more than one hue (Figure S4C). While Brainbow strategies are better suited to the CNS, they often still require complex immunolabeling to enhance the FP signal and fully exploit the available color diversity, thereby limiting their practical utility.^61^

### Chemogenetic neuromodulation of ing-scWAT sensory activity

**Figure 7:**
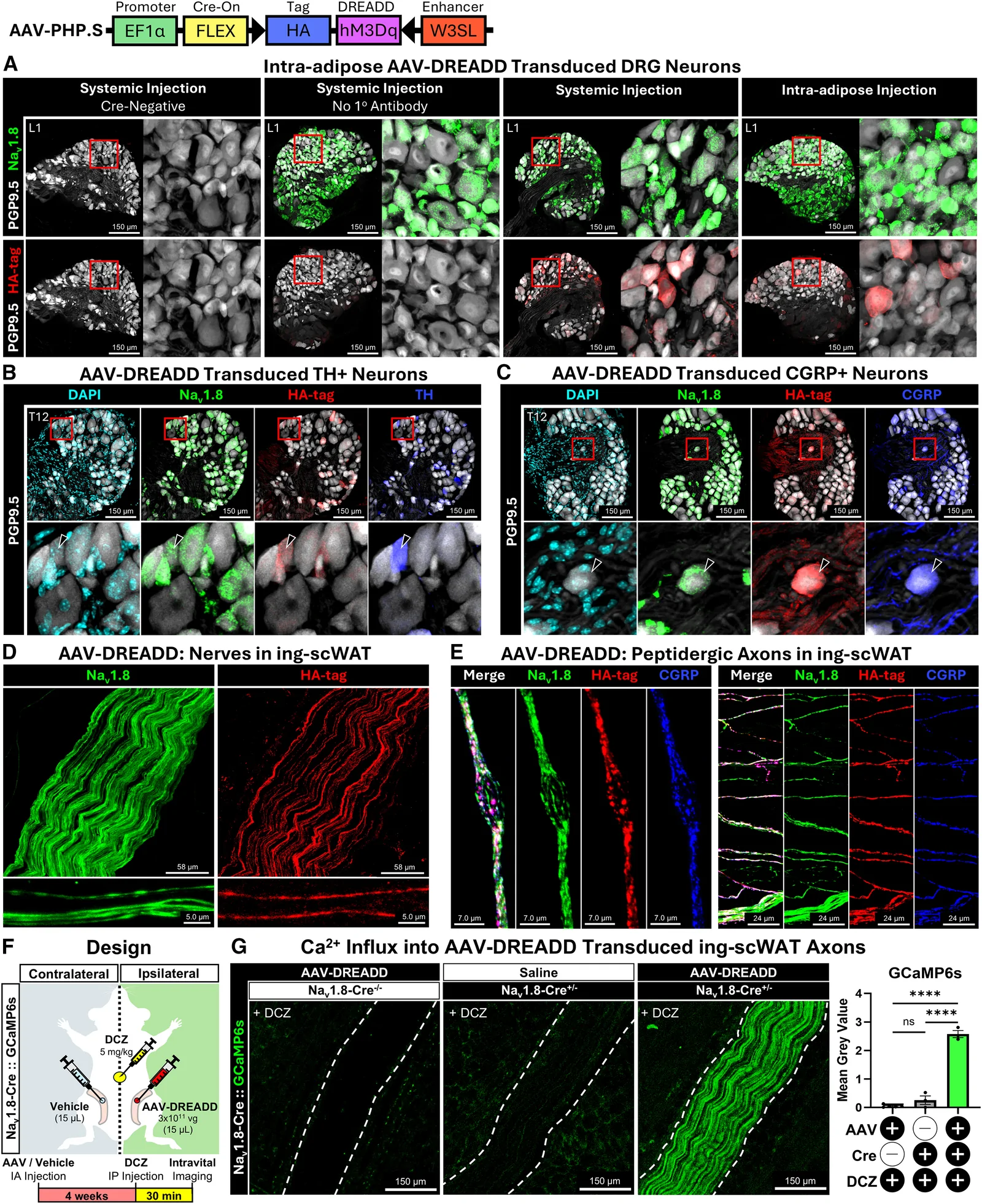
Intra-adipose delivery of AAV-DREADD into Na_v_1.8-Cre mice (A) AAV-PHP.S-EF1α-FLEX-HA-hM3Dq-W3SL (or “AAV-DREADD”) was injected into Na_v_1.8-Cre::ZsGreen1 mice (9-week-old) either systemically (1 × 10^12^ vg/mouse, r.o.) or via intra-adipose delivery (3 × 10^11^ vg/depot, i.a.). Thin-sectioned L1 DRGs were immunolabeled for the HA-tag transgene to verify transduction. Cre-negative mice and no-primary antibody controls were used to validate AAV-DREADD transduction. Red boxes indicate magnified regions. Nav1.8^+^ cells (green), AAV-DREADD (HA tag, red), neurons (PGP9.5, gray). (B) Co-expression of HA tag (red) and TH (blue) in thin-sectioned T12 DRGs (black arrow). Red boxes are digitally magnified for visualization. (C) Co-expression of HA tag (red) and CGRP (blue) in thin-sectioned T12 DRGs (black arrow). Red boxes are digitally magnified for visualization. (D) Intra-adipose AAV-DREADD-transduced sensory (Na_v_1.8^+^) axons in ing-scWAT. (E) Intra-adipose AAV-DREADD-transduced peptidergic (Na_v_1.8^+^/CGRP^+^) axons in ing-scWAT. (F) Experimental design for intravital calcium imaging of AAV-DREADD-transduced sensory axons. Na_v_1.8-Cre::GCaMP6s (*n* = 3, 10- to 14-weeks old) and GCaMP6s (*n* = 5; 13- to 14 weeks old) mice were injected unilaterally with AAV-DREADD (3 × 10^11^ vg/tissue, i.a.), and contralateral depots were injected with saline vehicle (*n* = 3). Deschloroclozapine (DCZ, 5 mg/kg, i.p.) was administered to each mouse 30 min prior to imaging. (G) Intravital imaging of ing-scWAT nerve bundles. Dashed lines indicate the ROIs selected for fluorescence intensity quantification. ^ns^p ≥ 0.05; ∗∗∗∗*p* < 0.0001, by one-way ANOVA. Error bars are ± SEM.

Finally, we applied our optimized Cre-dependent AAV approach to target nerves for future neuromodulatory studies. We noted that commercially available AAV vectors (e.g., from Addgene) favor tissue-specific promoters (such as hSyn1), which were unsuitable for our needs (Figure 3B). Consequently, we engineered our own vector, AAV-PHP.S-EF1α-FLEX-HA-hM3Dq, utilizing the EF1α promoter (“AAV-DREADD”) (Figure 7).

Key design criteria for this plasmid, based on the data presented herein, were as follows: (1) use of a ubiquitous promoter, (2) Cre-dependent expression, (3) incorporation of the excitatory DREADD receptor (hM3Dq), (4) a means to visualize viral transduction, and (5) a total cargo size below the 4.7 kb packaging limit. We selected the ubiquitous EF1α promoter over the CAG promoter to reduce genome size. We fused a hemagglutinin (HA) tag to hM3Dq for immunolabeling, all under a “Cre-on” FLEX switch. To further minimize the overall cargo size, we replaced the full-length Woodchuck hepatitis virus posttranscriptional regulatory element (WPRE) with W3SL, a truncated version comprising the essential regulatory and polyadenylation elements.^62^ As a result, our pAAV-DREADD construct measured only 4,167 bp (see vector map in Figure S5).

AAV-DREADD was administered to Na_v_1.8-Cre::ZsGreen1 mice either systemically (3 × 10^12^ vg/mouse, r.o.) or via i.a. injection (3 × 10^11^ vg/tissue). Immunolabeling against the HA tag revealed expression exclusively in Cre-positive mice (Figure 7A). Following AAV-DREADD injection into ing-scWAT, transduced neurons were retrogradely traced to DRG T11-L3 (Figure S6A). Co-labeling confirmed that TH^+^/Na_v_1.8^+^ and CGRP^+^/Na_v_1.8^+^ sensory neurons innervating ing-scWAT were successfully transduced (Figures 7B and 7C). Within ing-scWAT, a subset of axons in Na_v_1.8^+^ nerve bundles and parenchymal small peptidergic fibers also expressed the HA tag (Figures 7D and 7E).

To confirm the functional activity of the stimulatory DREADD transgene in ing-scWAT, we utilized Na_v_1.8-Cre::GCaMP6s mice (*n* = 3), which express the calcium indicator GCaMP6s in sensory neurons. AAV-DREADD (3 × 10^11^ vg, i.a.) was injected into the left ing-scWAT depot, while the contralateral depot received saline vehicle. Cre-negative GCaMP6s mice (*n* = 5) served as genetic controls. We first validated this intravital imaging approach using capsaicin, a potent agonist of TRPV1 receptors expressed by sensory nerves. TRPV1 is a cation channel that, upon activation, triggers an influx of Ca^2+^ into the cell. Accordingly, the vehicle-injected contralateral ing-scWAT depot was bathed in capsaicin (100 μL at 20 μg/μL), which induced a prominent calcium influx (Figure S6C). We, therefore, next tested whether the selective chemogenetic activation of AAV-DREADD-expressing sensory axons was sufficient to induce calcium signaling in ing-scWAT *in vivo*. The mice were administered the DREADD agonist deschloroclozapine (DCZ; 5 mg/kg, intraperitoneally [i.p.]) approximately 30 min prior to intravital imaging (Figure 7F). Significant GCaMP fluorescence was observed only within the nerve bundles of Cre-positive mice that received the AAV-DREADD (*p* < 0.0001; Figure 7G). No difference was observed between the Cre-negative and AAV-negative control groups. Collectively, these results demonstrated the effective and restricted depot-specific transduction and stimulation of subcutaneous adipose sensory nerves with a DREADD transgene.

## Discussion

This study systematically optimized i.a. AAV-mediated transgene delivery to the peripheral nerves. We started broadly with systemic injections and a ubiquitous promoter, and then iteratively narrowed our approach to target transduction of neural subtypes in individual adipose depots with an excitatory DREADD transgene. This is the first step toward addressing a significant gap in the gene therapy and neuromodulation literature, which has mostly overlooked peripheral nerves, including adipose tissue innervation.

The selection of an optimal vector is highly dependent on the tissue environment and the neuronal population of interest. One capsid notably absent from our testing was AAV6, which was previously used to transduce BAT sympathetic innervation in mice.^40^ AAV6 is highly tropic for neurons as well as muscles, among other cell types. AAV6 is transported retrogradely,^63^ which our data suggest is crucial for adequate transduction of axons in ing-scWAT. Addgene did not have an AAV6-CAG-tdTomato viral prep comparable to those we evaluated, so we did not assess this capsid. However, the literature suggests that AAV6 could be comparable to AAVrg in ing-scWAT, and this could be confirmed with future testing. Notably, one study reported successful transduction of BAT sympathetic axons using the hSyn1 promoter,^40^ while a separate study found that MaCPNS1 robustly labeled both sympathetic and sensory neurons when injected into BAT. In contrast, we found that the hSyn1 promoter and MaCPNS1 capsid were both too restrictive in ing-scWAT. This suggests a potential tissue-specific divergence in approaches to what works in BAT versus scWAT.

With this, we have demonstrated that systemic AAV delivery can be used to selectively transduce Nav1.8^+^ sensory axons across multiple adipose depots, including BAT, in adult mice, extending beyond depots previously shown to exhibit substantial sensory innervation. These findings expand the current understanding of adipose sensory targeting and highlight the broader potential of systemic AAV approaches for interrogating sensory-adipose communication. Note that because the transduction efficiency and specificity are likely to vary across depots, these results should be considered an initial framework that require depot-specific optimization and validation in future studies.

The ability to efficiently transduce adipose innervation opens avenues for studying energy balance and metabolic regulation. Given that the loss of adipose tissue innervation is implicated in obesity, aging, and diabetes, the vectors and strategies described herein provide a framework for developing therapeutic interventions targeting metabolic and neuropathic disorders. Tetbow was adapted for high-resolution, multicolor labeling of peripheral sensory axons in adipose tissue, enabling reliable discrimination of adjacent fibers that is not readily achievable with Brainbow-based approaches. This application provides a versatile framework for future studies of adipose tissue innervation, axonal remodeling, and neuro-adipocyte interactions across the physiological and disease states. Our optimized approach to deliver excitatory DREADDs to neural subtypes in ing-scWAT, for example, demonstrates how these methods can be used in the future for precise neuromodulation. In this vein, we are using these methods to target adipose sensory nerves for short hairpin RNA (shRNA)-mediated gene knockdown. Together, this offers a promising tool for investigating axonal subtype functions and determining the effects of altering nerve activity, without the same degree of off-target effects as tissue denervation or whole-body knockdown approaches.

The inherent weakness in transgene delivery to axons calls for further methodological optimization, particularly as post-translational modifications like farnesylation, which enhances membrane binding of FPs,^19^ might offer additional avenues to improve the labeling and visualization of nerve fibers. The “parent capsid” of PHP.S and AAV9 offers broad tropism and an apparent ability to co-op Rab-positive endosomes for axonal trafficking,^64^^,^^65^ which likely enhances retrograde transport to the soma, thereby improving peripheral nerve transduction. PHP.S differs from AAV9 by a short peptide insertion in a surface-exposed capsid loop,^18^ which could contribute to improved uptake into peripheral neurons, improved intracellular trafficking, and/or reduced degradation; this remains speculative and has not been directly tested. Further investigation of the mechanisms underlying the efficient peripheral axon transduction by PHP.S, including the identification of specific receptors for uptake, is necessary to inform future capsid engineering efforts. Likewise, the enhancer AAV approach has been developed exclusively to target neurons in the CNS, and the potential for enhancer-AAVs to further enable selective targeting of specific PNS neuron subtypes warrants investigation.

The pAAV 4.7 kb cargo size restriction also imposes limitations on study design. Here, we would have preferred to use the slightly stronger CAG promoter over EF1α for our AAV-DREADD, but we needed to reduce the cargo size. Developments such as mini-DREADDs have reduced DREADD packaging size by up to 35% without affecting the receptor properties.^66^ Incorporating these advances would greatly expand the utility of this approach, especially alongside the development of enhancer AAVs capable of selectively targeting defined peripheral neuron populations.

Overall, our study establishes a framework for the AAV-mediated transduction of adipose tissue innervation and highlights the nuanced interplay between vector choice, promoter selection, and tissue-specific factors. This work lays the groundwork for an improved understanding of adipose tissue innervation and for the development of targeted therapeutic interventions for metabolic and neuropathic diseases.

### Limitations of the study

This study was designed as an iterative optimization framework, progressing from broad exploratory screening to targeted validation, and several limitations should be considered when interpreting the findings and/or applying them to future studies. Early-stage capsid and promoter comparisons were performed with low sample sizes and relied on the qualitative assessment of fluorescent transgene expression rather than formal quantification. These experiments were intentionally structured as “go/no-go” screening steps to identify conditions that achieved sufficiently robust and interpretable transduction for downstream applications. Given the large number of parameters, including capsid, promoter, delivery route, viral titer, and vector design, this approach prioritized feasibility and experimental tractability over exhaustive quantification. However, this method necessarily limited statistical power and precluded definitive comparisons across all conditions. Accordingly, these results should be interpreted as directional to inform later cohort assessments and serve as a practical resource for guiding experimental design, rather than a definitive framework.

Quantitative assessment of transgene localization and subtype-specific transduction in adipose tissue is inherently challenging. Subcutaneous WAT is a heterogeneous, densely innervated tissue with interwoven parenchymal, neurovascular, and mixed-nerve bundle structures containing both myelinated and non-myelinated fibers. These features are continuous rather than discrete, making segmentation subjective and highly sensitive to the imaging depth, thresholding, and sampling. Additionally, commonly used markers (e.g., TH) lack strict specificity in adipose tissue, with overlapping expression across neuronal subtypes. As a result, formal co-localization or compartment-specific quantification would risk classification bias and overinterpretation, which we sought to avoid as we demonstrated proof-of-concept data.

Importantly, AAV-mediated gene delivery is highly sensitive to experimental variables. Transduction efficiency and tropism are influenced by the capsid design, delivery route, viral titer, cargo size, promoter and enhancer elements, tissue architecture, and even technical factors such as virus handling. These effects can be further modulated by species- and strain-specific differences,^67^^,^^68^ making direct comparisons across studies inherently challenging. Alterations in any aspect of the experimental design can critically influence outcomes, and published findings should be interpreted accordingly. For example, reporting the number of transduced cells or percentages of overlap and co-expression within the DRGs and/or SChGs is often necessary for interpreting the results of individual experiments. Still, these data are highly specific to the experimental conditions described in each singular study. Altering any of the abovementioned factors can significantly change these outcomes, limiting the direct translation of these results to other experimental setups, which is likely the reason why reports vary so drastically between studies. We recommend that the use of this approach be preceded by a validation study similar to what we have done here, so that any changes to viral production, vector design, delivery route, mouse model, etc. are accounted for in the interpretation of findings.

Finally, our Cre-dependent strategy for achieving neuronal subtype-specific expression is constrained by the availability and specificity of Cre-driver lines. While effective in the context of Na_v_1.8-Cre mice, off-target and leaky recombination observed in other lines (e.g., TH-Cre) highlight the need for rigorous validation and limits broader applicability. Additionally, reporter signal dynamics posed technical constraints: in Cre-dependent approaches, signal often approached autofluorescence thresholds, whereas in Cre-independent approaches, widespread non-neuronal expression obscured neural labeling, complicating whole-tissue quantification.

Despite these constraints, combining rapid screening with focused validation allowed us to identify AAV strategies that are robust and experimentally useful for targeting adipose innervation. Future work incorporating larger cohorts, standardized quantification, and improved tools for neuronal specificity is essential to refine and generalize these methods.

## Resource availability

### Lead contact

Requests for further information, resources, and reagents should be directed to and will be fulfilled by the lead contact, Dr. Kristy L. Townsend (kristy.townsend@osumc.edu).

### Materials availability

Plasmids generated in this study have been deposited with Addgene, plasmid #240285. Plasmids encoding engineered capsids are available as pAAV plasmid at Addgene: PHP.S (#103006), MaCPNS1 (#185136), and MaCPNS2 (#185137). AAV vectors that were provided by the CPP NIH BRAIN Initiative Armamentarium Vector Core are available for purchase upon request and were produced using plasmids available from Addgene.

### Data and code availability

• All data reported in this paper will be shared by the lead contact upon request.
• This paper does not report original code.
• Any additional information required to reanalyze the data reported in this paper is available from the lead contact upon request.

## Acknowledgments

The authors thank Tianyi Tao (OSU), Dr. Holly Sucharski-Argall (OSU), Akshaykumar Ganesh (OSU), Fernando Garcia (CPP), Kalif Johnson (CPP), and Damien Wolfe (CLOVER) for technical assistance. We thank Drs. Andrea Tedeschi (OSU) and Magdalena Blaszkiewicz (OSU) for helpful discussions and assistance with AAVs. We also thank Dr. Lori Zeltser (CUIMC) for providing CTB tracing methods. Some figures were made, in part, with assets from BioRender. J.W.W. was funded by a Presidential Graduate Fellowship from The Ohio State University. K.L.T. was supported by a W.M. 10.13039/100000888Keck Foundation Award (sponsor award ID: 995699|MR|9181) and start-up funding from The Ohio State University. Research reported in this publication was supported by the 10.13039/100000025National Institute of Mental Health of the 10.13039/100000002National Institutes of Health under award number U24MH131054. The content is solely the responsibility of the authors and does not necessarily represent the official views of the National Institutes of Health.

## Author contributions

Conceptualization, K.L.T.; investigation, J.W.W., L.M.L. G.G., and G.M.; methodology, J.W.W., T.F.S., and A.D.S.; formal analysis, J.W.W.; validation, J.W.W.; visualization, J.W.W.; writing – original draft and revision, K.L.T. and J.W.W.; writing – review & editing, L.M.L., T.F.S., and A.D.S.; resources, A.P.V. and A.D.S.; project administration, K.L.T.; supervision, K.L.T. and J.W.W.; funding acquisition, K.L.T. and J.W.W.

## Declaration of interests

The authors declare no competing interests.

## Declaration of generative AI and AI-assisted technologies in the writing process

During the preparation of the first draft of this work, the authors used ChatGPT and Grammarly to improve grammar and readability. After initial use of these tools/services, the authors all wrote, reviewed, and edited the content themselves and take full responsibility for the content of the published article.

## STAR★Methods

### Key resources table

**Table:** 

REAGENT or RESOURCE	SOURCE	IDENTIFIER
**Antibodies**		

Rabbit anti-TH	EMD Millipore	Cat#: ab152; RRID: AB_390204
Rabbit anti-CGRP	Cell Signaling	Cat#: 14959; RRID: AB_2798662
Rabbit anti-SYN1	Cell Signaling	Cat#: 14959; RRID: AB_2798662
Rabbit anti-HA	Cell Signaling	Cat#: 3724; RRID: AB_1549585
Mouse anti-2H3	DSHB	Cat#: 2H3; RRID: AB_531793
Mouse anti-SV2	DSHB	Cat#: SV2; RRID: AB_2315387
Rabbit anti-GFP (conj. AF488)	Invitrogen	Cat#: A21311; RRID: AB_221477
Rabbit anti-PGP9.5 (conj. AF488)	Abcam	Cat#: ab302664; RRID: N/A
Rabbit anti-PGP9.5 (conj. AF647)	Abcam	Cat#: ab196173; RRID: N/A
Rabbit anti-HA (conj. AF594)	Cell Signaling	Cat#: 45346; RRID: AB_2924897
Goat anti-Rabbit IgG (H + L) AF594+	Invitrogen	Cat#: A32740; RRID: AB_2762824
Goat anti-Rabbit IgG (H + L) AF647+	Invitrogen	Cat#: A32733; RRID: AB_2633282
Goat anti-Mouse IgG1 AF647	Invitrogen	Cat#: A21121; RRID: AB_2535804

**Bacterial and virus strains**		

pAAV EF1α-FLEX-hM3D(Gq)-HA-WPRE3-SV40pA	Addgene	Cat#: 59462-AAV1; This paper
pUCmini-iCAP-PHP.S	Addgene	Cat#: 103006
pUCmini-iCAP-AAV.MaCPNS1	Addgene	Cat#: 185136
pUCmini-iCAP-AAV.MaCPNS2	Addgene	Cat#: 185137
AAV1-CAG-tdTomato	Addgene	Cat#: 59462-AAV1
AAV5-CAG-tdTomato	Addgene	Cat#: 59462-AAV5
AAV9-CAG-tdTomato	Addgene	Cat#: 59462-AAV9
AAV9-hSyn-EGFP	Addgene	Cat#: 50465-AAV9
AAV9-EF1α-EGFP	Addgene	Cat#: 105547-AAV9
AAVrg-CAG-tdTomato	Addgene	Cat#: 59462-AAVrg
AAV-PHP.S-CAG-tdTomato	Addgene	Cat#: 59462-PHP.S
AAV-PHP.S-CAG-FLEX(tdTomato)	Addgene	Cat#: 28306-PHP.S
AAV-PHP.S-CAG-DIO(tTA)	CPP Vector Core	Cat#: N/A
AAV-PHP.S-TRE-DIO(mTurqoise2)	CPP Vector Core	Cat#: N/A
AAV-PHP.S-TRE-DIO(mNeonGreen)	CPP Vector Core	Cat#: N/A
AAV-PHP.S-TRE-DIO(tdTomato)	CPP Vector Core	Cat#: N/A
AAV-PHP.S-EF1α-FLEX(HA-hM3Dq)	CPP Vector Core	Cat#: N/A; This paper
AAV-MaCPNS1-CAG-EGFP	CPP Vector Core	Cat#: N/A

**Chemicals, peptides, and recombinant proteins**		

Paraformaldehyde	Sigma-Aldrich	Cat#: P6148
10X PBS Solution	Teknova	Cat#: P0496
Sodium Azide	Sigma-Aldrich	Cat#: S2002
Bovine Serum Albumin	Sigma-Aldrich	Cat#: A4503
Triton X-100	Bio-Rad Laboratories	Cat#: 1610407
DAPI, dilactate	Sigma-Aldrich	Cat#: D9564
Heparin Sodium Salt from Porcine Mucosa	Sigma-Aldrich	Cat#: H3393
EMS Glycerol Mounting Medium With DABCO	Electron Microscopy Sciences	Cat#: 17989-5
0.9% Sodium Chloride Injection, USP	Fisher Scientific	Cat#: NC9054335
Tissue-Tek O.C.T. Compound	Sakura	Cat#: 4583
Target Retrieval Solution	Agilent	Cat#: S169984-2
Ethanol	Sigma-Aldrich	Cat#: E7023
Acetone	Fisher Scientific	Cat#: A18-4
Tween 20	EMD Millipore	Cat#: 655205
IHC Blocking Reagent	EMD Millipore Corp	Cat#: 20773
20X Rinse Buffer	EMD Millipore Corp	Cat#: 20845
Antibody Diluent	Dako	Cat#: S0809
Light Diagnostics Mounting Fluid (non-permanent)	Sigma-Aldrich	Cat#: 5013
Cholera Toxin Subunit B (CTB) (conj. AF647)	ThermoFisher	Cat#: C34778
Deschloroclozapine	Tocris	Cat#: 7193
Capsaicin	Sigma	Cat#: M2028
Zamboni Fixative	Fisher Scientific	Cat#: NC9335034

**Experimental models: Organisms/strains**		

Mouse: C57BL/6J	The Jackson Laboratory	Stock#: 000664
Mouse: Na_v_1.8-Cre	The Jackson Laboratory	Stock#: 036564
Mouse: NET-Cre	The Jackson Laboratory	Stock#: 037882
Mouse: TH-Cre	The Jackson Laboratory	Stock#: 008601
Mouse: CMV-Cre	The Jackson Laboratory	Stock#: 006054
Mouse Brainbow3.2 (Line 7)	The Jackson Laboratory	Stock#: 021227
Mouse: Ai6(RCL-ZsGreen1)	The Jackson Laboratory	Stock#: 007906
Mouse: Ai9(RCL-tdTomato)	The Jackson Laboratory	Stock#: 007909
Mouse: Ai96(RCL-GCaMP6s)	The Jackson Laboratory	Stock#: 028866

**Recombinant DNA**		

pAAV-EF1α-FLEX-HA-hM3Dq	VectorBuilder	Cat#: N/A

**Software and algorithms**		

FIJI	Schindelin et al.^69^	https://fiji.sc/
Leica Application Suite X	Leica Microsystems	https://www.leica-microsystems.com/products/microscope-software/p/leica-las-x-ls/
Prism (v10)	GraphPad	https://www.graphpad.com/
Cellpose	Stringer et al.^70^ Pachitariu and Singer^71^	https://github.com/MouseLand/cellpose
PowerPoint	Microsoft	https://www.microsoft.com/en-us/microsoft-365/powerpoint

**Other**		

75 × 51 mm Glass Slide, 1.2 mm thick	Electron Microscopy Sciences	Cat#: 71862-01
Cover glass, 48 × 60 mm, 1.5 thick	Brain Research Laboratories	Cat#: 4860-1.5D
25 × 75 × 1.0 mm Superfrost Plus™ Microscope Slides	Fisher Scientific	Cat#: 1255015
Cover Glass, 22 × 50 mm, #1.5	VWR	Cat#: 16004-336
47 mm diameter Glass bottom dish	WilCo Wells	Cat#: GWSB-5030
5 cm Wide Binder Clips	ACCO	Cat#: 72102
6 well plate	VWR	Cat#: 10861-696
Leica Stellaris 5 Confocal Microscope	Leica Microsystems	Cat#: NA
Orbital Shaker	VWR	Cat#: 89032-100
0.5 mL U-100 Insulin Syringe, 28G	BD	Cat#: 329461
0.3 mL U-100 Insulin Syringe, 31G	McKesson	Cat#: 942674
Low Protein Binding Microcentrifuge Tubes, 1.5 mL	ThermoFisher	Cat#: 90410

### Experimental model and study participant details

All procedures and handling of animals were performed in accordance with The Ohio State University’s Institutional Animal Care and Use Committee (IACUC) to comply with the guidelines of the PHS Policy on Humane Care and Use of Laboratory Animals and Guide for the Care and Use of Laboratory Animals. The Ohio State University’s IACUC approved this study under protocol 2021A00000004. All mice were obtained from The Jackson Laboratory (Bar Harbor, ME) and/or bred at our mouse facility at The Ohio State University. Mice were housed initially as 4–5 in a cage in a climate-controlled vivarium with a 12/12 h light/dark cycle and *ad libitum* access to food and water. Mice were euthanized by CO_2_ asphyxiation with cervical dislocation as a secondary confirmation of death. All mice used in experiments were between 8 and 33 weeks of age.

*C57BL/6J (JAX #000664);* male and female C57BL/6J mice were used for optimizing AAV injections when Cre recombinase activity was not required. *Na*_*v*_*1.8-Cre (JAX #036564);* male and female Na_v_1.8-Cre mice were used to drive transgene expression in sensory neurons. NET-Cre (*JAX #037882);* male and female NET-Cre mice were used to drive transgene expression in cells that express norepinephrine transporter. *ZsGreen1* or *Ai6* (*JAX #007906);* male and female ZsGreen1 mice were bred to Na_v_1.8-Cre and NET-Cre mice. TH-Cre (*JAX #008601)*; male and female TH-Cre mice used to drive recombination in cells that express tyrosine hydroxylase. *tdTomato or Ai9 (JAX #007909);* male and female mice were bred to TH-Cre and Na_v_1.8-Cre mice. *Brainbow3.*2 (JAX #021227); male and female mice express brainbow transgenes under control of Thy1.2 promoter and were bred to CMV-Cre mice. CMV-Cre (*JAX #*006054); male and female mice were used to drive ubiquitous transgene expression and bred to Brainbow3.2 mice, which restricted expression to Thy1.2^+^ cells. *GCaMP6s or Ai96 (JAX #028866);* male and female mice were bred to Na_v_1.8-Cre mice.

### Method details

#### Plasmid and viral production/packaging

pAAV-DREADD used in this study was generated with VectorBuilder (Figure S5). Viral packaging was performed using triple-transfection of HEK cells and iodixanol gradients for purification as previously described.^72^ Table S1 provides a detailed list of the vectors used in this study and their corresponding viral titers, administration routes, and promoter sequences.

AAV genome titers were quantified using droplet digital PCR (ddPCR) on the Bio-Rad QX system with a droplet generator. Viral preparations were first treated with DNase I (1–2 U per 10–20 μL sample, 37°C for 30 min) to remove unpackaged DNA, followed by heat inactivation at 65°C for 10 min. Samples were serially diluted in nuclease-free water to achieve concentrations within the dynamic range of the assay (typically >10^6^-fold dilutions). Each ddPCR reaction contained ddPCR Supermix for Probes (no dUTP), ITR-specific primers, SYBR green, diluted template, and nuclease-free water to volume. Droplets were generated using a DG8 cartridge and droplet generation oil and subsequently transferred to a 96-well PCR plate for thermal cycling. Cycling conditions consisted of 95°C for 10 min, followed by 40 cycles of 94°C for 30 s and 60°C for 60 s with a ramp rate of 2°C/s, and a final enzyme deactivation step of 98°C for 10 min before holding at 4°C. Droplets were read on the Bio-Rad droplet reader, and data were analyzed in QuantaSoft using no-template controls to set thresholds. Final viral genome titers were calculated from the copies/μL output of QuantaSoft, corrected for input volume and dilution factor, and reported as viral genomes per milliliter (vg/mL).

#### AAV injection

*Systemic Injection:* 20–150 μL of AAV (dependent on required viral titer) was loaded into a 0.5 cc insulin syringe (28G). Mice were anesthetized with 5% isoflurane with oxygen flow at 1 LPM, and AAV was injected into the retro-orbital (r.o) sinus. If more than 100 μL of AAV was required, half of the total volume was injected behind each eye. Virus incubated for 4 weeks before tissues were collected.

*Intra-adipose Transdermal Microinjection (i.a.):* mice were anesthetized with 2–5% isoflurane at 1 LPM. The ing-scWAT depot was located beneath the skin by gentle palpation of the mouse’s flank. Ing-scWAT was pinched and pulled slightly up and away from the peritoneum for injections. 5–50 μL of AAV (dependent on required viral titer) was loaded into a 0.3 cc insulin syringe (31G) and injected into ing-scWAT by intra-adipose transdermal microinjection (5 μL per injection, 1–10 injections depending on total viral particles to be injected).

For all experiments, tissues were collected four weeks post-injection, sufficient for AAV-mediated transgene expression to reach stable, near-maximal levels *in vivo*, consistent with prior reports of expression plateauing by 4–6 weeks across tissues and serotypes and our own observations in ing-scWAT.^37^^,^^73^^,^^74^^,^^75^ All AAVs were stored at −80°C and thawed only once prior to injection.

#### Cholera toxin subunit B (CTB)

A 0.2% working solution was prepared by dissolving 100 μg of CTB (Recombinant, Alexa Fluor 647 Conjugate, Thermofisher, Cat# C34778) in 50 μL sterile saline. Mice were anesthetized with 2–5% isoflurane at 1 LPM. The ing-scWAT depot was located beneath the skin by palpation and was pinched and pulled away from the peritoneum for transdermal microinjections. Ing-scWAT received four 5 μL injections four days prior to tissue harvest, which was chosen to ensure maximal tracing efficiency.^76^

#### Whole mount immunofluorescence

*Adipose Tissue:* Intact inguinal and axillary scWAT, BAT, and pgWAT depots were excised from male and female mice, fixed overnight in 2% PFA at 4°C, and processed following the Z-depth reduction method described previously^50^ with accompanying protocol.^77^

*Neuromuscular Junctions (NMJ):* The medial gastrocnemius was excised and fixed for 2 h in 2% PFA at 4°C. Following two 1-h washes in 1X PBS, tissues were processed for immunostaining as previously described.^36^

*Sciatic Nerve (SciN):* ∼1 cm section of the sciatic nerve was excised from each mouse and fixed for 2 h in 2% PFA at 4°C. Tissues received two 1-h washes in 1X PBS and were mounted on glass slides with a glycerol-based mounting media (EMS, Cat#17989). A coverslip was added, and the slides were sealed with nail polish.

*Liver, Sternum, and Testes:* Whole organs were excised from mice and fixed overnight in 2% PFA at 4°C. The following day they received two 1-h washes in 1X PBS. Organs were placed onto a glass-bottom dish (WilCo, Cat#:GWSB-5030) containing 1X PBS for imaging.

*Dorsal Root Ganglia (DRG) and Sympathetic Chain Ganglia (SChG):* Ganglia were excised, fixed in 4% PFA at 4°C for 2 h, and washed twice in 1X PBS for 2 h. Ganglia were incubated in 1X PBS/2.5% BSA/1% Triton X-100 for 3 days at 4°C. If immunolabeling was required, ganglia were incubated in primary antibody for 5 days at 4°C, received four 1-h washes in 1X PBS, and incubated in secondary antibody for 2 days at 4°C. Tissues were washed four times in 1X PBS, 1 h each. Tissues were placed onto glass slides with 30 μL of glycerol-based mountant, and a glass coverslip was applied and sealed with nail polish.

See Table S2 for a comprehensive list of all antibodies.

#### Thin section immunofluorescence

*DRGs:* were excised bilaterally and immediately fixed in Zamboni Fixative (Newcomer Supply, Cat#1459A) for 1 h at room temperature, after which tissues received one wash in 1X PBS at room temperature. DRGs were transferred to fresh 1X PBS and incubated overnight at 4°C. The following day, DRGs were moved into 30% sucrose overnight at 4°C for cryo-protection before embedding in O.C.T. compound (Tissue-Tek, Cat#4583). DRGs were sectioned at 14 μm onto glass slides and stored at −20°C until immunostaining.

*The liver (left lobe):* was excised and fixed overnight in 10% buffered formalin (Fisher Scientific, 23–245685). Liver tissue was washed in 1X PBS before cryo-protection by 30% sucrose. Tissues were embedded in O.C.T. compound, sectioned at 14 μm thick onto glass slides, and stored at −20°C until immunostaining.

For immunostaining, slides were submerged in ice-cold acetone for 15 min, followed by two 5-min washes in 1X Millipore Rinse Buffer (Millipore, Cat#20845). Next, slides were submerged for 20 min in 0.5% Tween 20 in 1X PBS, followed by two 5-min washes in rinse buffer. Slides were then immersed in Target Retrieval Solution (Agilent, Cat#S169984-2) and received two more 5-min washes in rinse buffer. Hydrophobic barriers were drawn around each tissue, and a drop of IHC Select Blocking Reagent (EMD Millipore, Cat#20773) was added to each tissue and incubated at 37°C for 20 min. Primary antibodies were diluted in Antibody Diluent (Agilent, Cat#S080983-2). 100 μL of antibody solution was added to each tissue and incubated overnight at 4°C. The next day, slides were washed twice for 10 min in rinse buffer and incubated in secondary antibody solution for 10 min at room temperature. Slides were washed twice for 10 min in rinse buffer and two more washes in deionized H_2_O for 5 min each. Slides were submerged in DAPI 100 ng/mL in H_2_O (Sigma-Aldrich, Cat#D9564) for 5 min at room temperature, followed by two more 5-min washes in deionized H_2_O. Slides were coverslipped and sealed for imaging.

See Table S2 for a comprehensive list of all antibodies.

#### Confocal microscopy

Micrographs were captured on a Leica Stellaris 5, laser scanning confocal microscope. The field of view was scanned bidirectionally at 600 Hz. Pinhole Airy set at 1.00 AU. Photons were detected with adjustable Power HyD S detectors, and scanned lines were averaged 2–5 times. Fluorescent labels were excited with a diode 405 nm laser (DAPI) and an adjustable white light laser (AF488, AF555, AF594, AF647). Excitation and emission spectra were tuned specifically for each fluorophore or group of fluorophores to eliminate crosstalk. Multiple channels were scanned sequentially. Objectives included: HC PL APO 10x/0.40 CS2, HC PL APO 40x/1.30 OIL CS2, and HC PL APO 63x/1.40 OIL CS2. Confocal zoom was applied to increase magnification further when necessary. Entire tissues were visually scanned, and multiple representative images were captured of each. Pixel resolution per field of view was either 1024 x 1024 or 2048 x 2048. All micrographs are displayed as z-maximum intensity projections. Tiling of projected z-stacks was performed to display entire adipose depots. LUTs were adjusted to improve structure visualization. All imaging parameters were kept consistent between groups when making direct comparisons. DRG and SChG cell bodies were segmented and counted using Cellpose.^70^^,^^71^ Image processing was performed in Leica Application Suite X, Fiji,^69^ and Microsoft PowerPoint.

#### Intravital calcium imaging

Na_v_1.8-CregCAMP6s mice received unilateral microinjection of AAV-DREADD (15μL, 3x10^11^ vg/tissue, i.a.) into ing-scWAT and saline vehicle in the contralateral depot. Following a 4-week transduction period, the mouse received an IP injection of deschlorclozapine (DCZ, 5 mg/kg) and was prepped for intravital imaging 30 min after the DCZ injection. The mouse was anesthetized with 2.5% isoflurane at 1 LPM. The skin above each inguinal scWAT depot was shaved, and a surgical window was made to visualize the depot. The mouse was placed on a 75 × 51mm glass slide (EMS, Cat# 71862-01), with the exposed ing-scWAT facing downward toward the 10× objective. See Figure S6B for a diagram. Imaging was performed as described above except that the PinholeAiry was set to 5.00 AU.

### Quantification and statistical analysis

Statistical calculations for determining significance were made in Prism (GraphPad Software). Indicators of statistical significance were unpaired two-tailed Student’s t-tests and one-way ANOVAs with Tukey’s correction for multiple comparisons. All error bars are SEMs. When *p*-value is otherwise not directly stated: ^ns^p > 0.05, ∗*p* < 0.05, ∗∗*p* < 0.01, ∗∗∗*p* < 0.001, ∗∗∗∗*p* < 0.0001.

## Supplemental information

Document S1. Figures S1–S6 and Tables S1 and S2

Document S2. Article plus supplemental information
