# Supplementary material for: Targeting the peripheral nervous system in adipose tissue with engineered AAV vectors
Source: Cell Rep Methods. 2026 Jul 31;6(9):101542. doi: 10.1016/j.crmeth.2026.101542 (PMC13615495; doi:10.1016/j.crmeth.2026.101542)
Supplement: Supplementary file 1 [file mmc1.pdf]

Cell Reports Methods, Volume *6*

Supplemental information

Targeting the peripheral nervous system in adipose tissue with engineered AAV vectors

Jake W. Willows, Lindsey M. Lazor, Gilian Gunsch, Gargi Mishra, Andrew P. Villa, Timothy F. Shay, Andrew D. Steele, and Kristy L. Townsend

Supplemental Figure S1: Peripheral tissues transduced by systemic injection of AAVs, related to Figure 1.

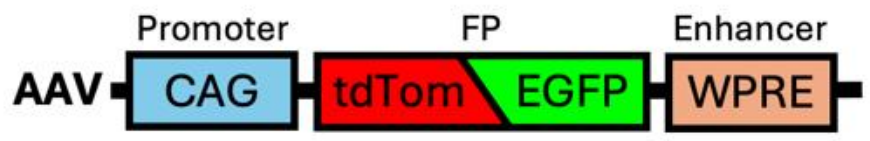

A

AAV Capsid Details

Capsid	Source	Transport
**AAV1**	Naturally occurring	Anterograde, transsynaptic
**AAV5**	Naturally occurring	Anterograde
**AAV9**	Naturally occurring	Bidirectional, transsynaptic
**AAVrg**	Engineered from AAV2	Retrograde
**AAV-PHP.S**	Engineered from AAV9	Presumed bidirectional
**AAV-MaCPNS1**	Engineered from AAV9	Presumed bidirectional
**AAV-MaCPNS2**	Engineered from AAV9	Presumed bidirectional

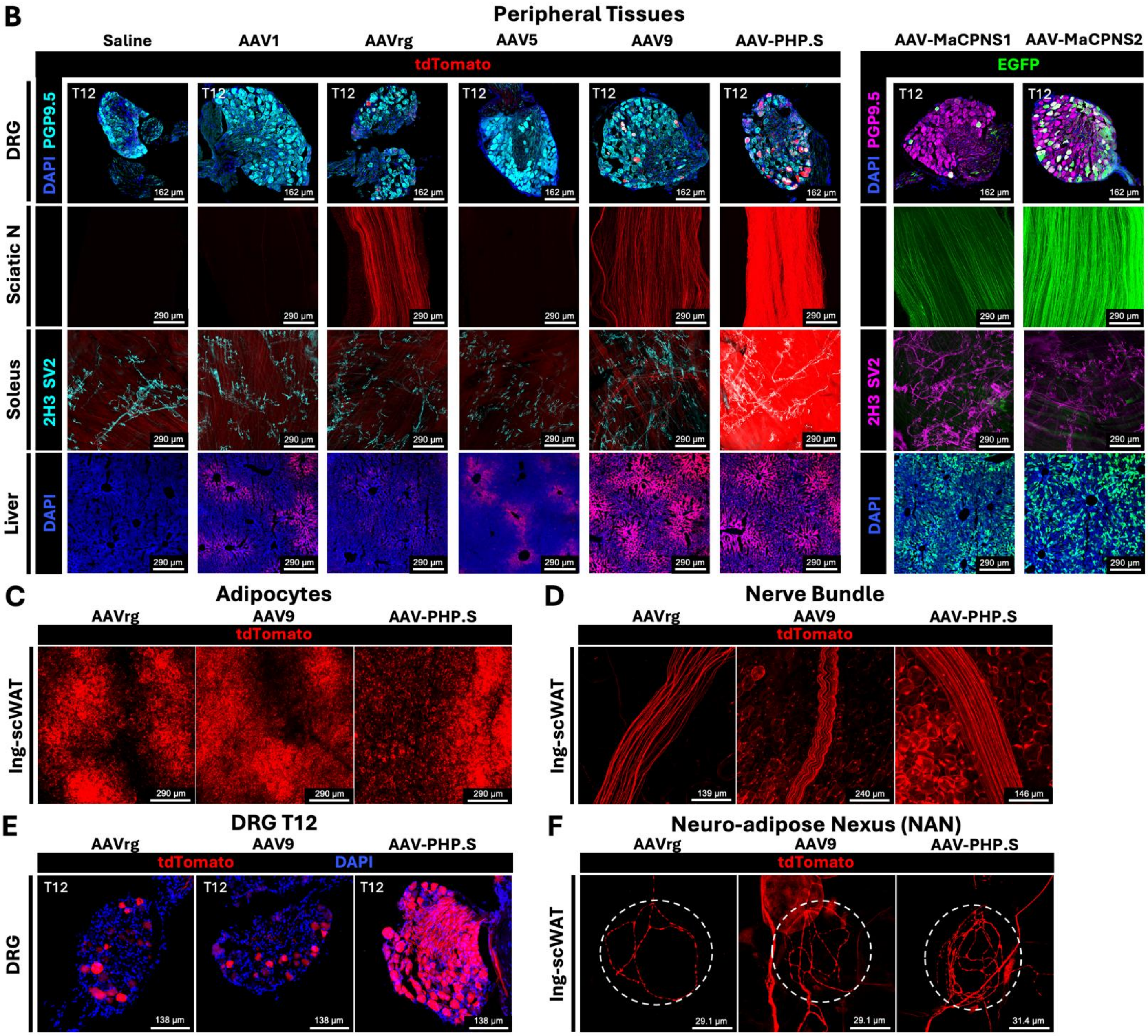

G

Relative Comparison of AAV Serotypes

	Ing-scWAT Nerve Bundles		Ing-scWAT Neurovascular		Ing-scWAT Parenchyma		NAN	Sciatic N	Soleus NMJs	Liver	DRG (T12)
	TH	CGRP	TH	CGRP	TH	CGRP					
**AAV1**	-	-	-	-	-	-	-	+	-	++	-
**AAVrg**	+++	+++	+++	+++	+++	+++	+++	++	-	+	++
**AAV5**	-	-	-	-	-	-	-	-	-	++	-
**AAV9**	+++	+++	+++	+++	+++	+++	+++	++	-	+++	++
**AAV-PHP.S**	+++	+++	+++	+++	+++	+++	+++	+++	-	+++	+++
**AAV-MaCPNS1**	-	+	-	-	-	-	-	++	-	+++	++
**AAV-MaCPNS2**	++	+++	-	+	-	+	-	+++	-	+++	+++

Supplemental Figure S1: Peripheral tissues transduced by systemic injection of AAVs, related to Figure 1.

- **(A)** List of AAV serotypes/capsids used in this paper, whether they are naturally occurring, and specifics on transport direction.
- **(B)** Male and female C57BL/6J mice (16-20-wk-old) were systemically injected with one of seven AAV capsids ($1x10^{12}$ vg/mouse, r.o.) and compared against a saline-injected control mouse; AAV-CAG-tdTomato/EGFP-WPRE. Fluorescent protein expression (tdTomato, red; EGFP, green) was compared in the T12 DRG (PGP9.5, cell bodies in cyan/magenta; DAPI, nuclei in blue), sciatic nerve, the neuromuscular junctions of the soleus (pre-synaptic terminal labeled by 2H3 and SV2 in cyan/magenta), and within the liver (DAPI nuclei in blue).
- **(C)** Replication of the three most promising capsids in male C57BL/6J mice (23-wk-old): AAVrg, AAV9, and PHP.S. Fluorescent transgene expression of ing-scWAT adipocytes.
- **(D)** Replication of transgene expression in ing-scWAT nerve bundles.
- **(E)** Replication of transgene expression within T12 DRG (nuclei, DAPI).
- **(F)** Identification that all three capsids successfully transduce the neuro-adipose nexus (NAN), outlined by dashed circles.
- **(G)** Relative observed differences in fluorescent transgene intensity and transduction efficiency of ing-scWAT and other tissues when injected systemically with AAV-CAG-tdTomato/EGFP-WPRE. +++ highest expression, ++ medium expression, + low expression, - no expression.

Supplemental Figure S2: Promoter sequences and titration for systemic delivery, related to Figure 1.

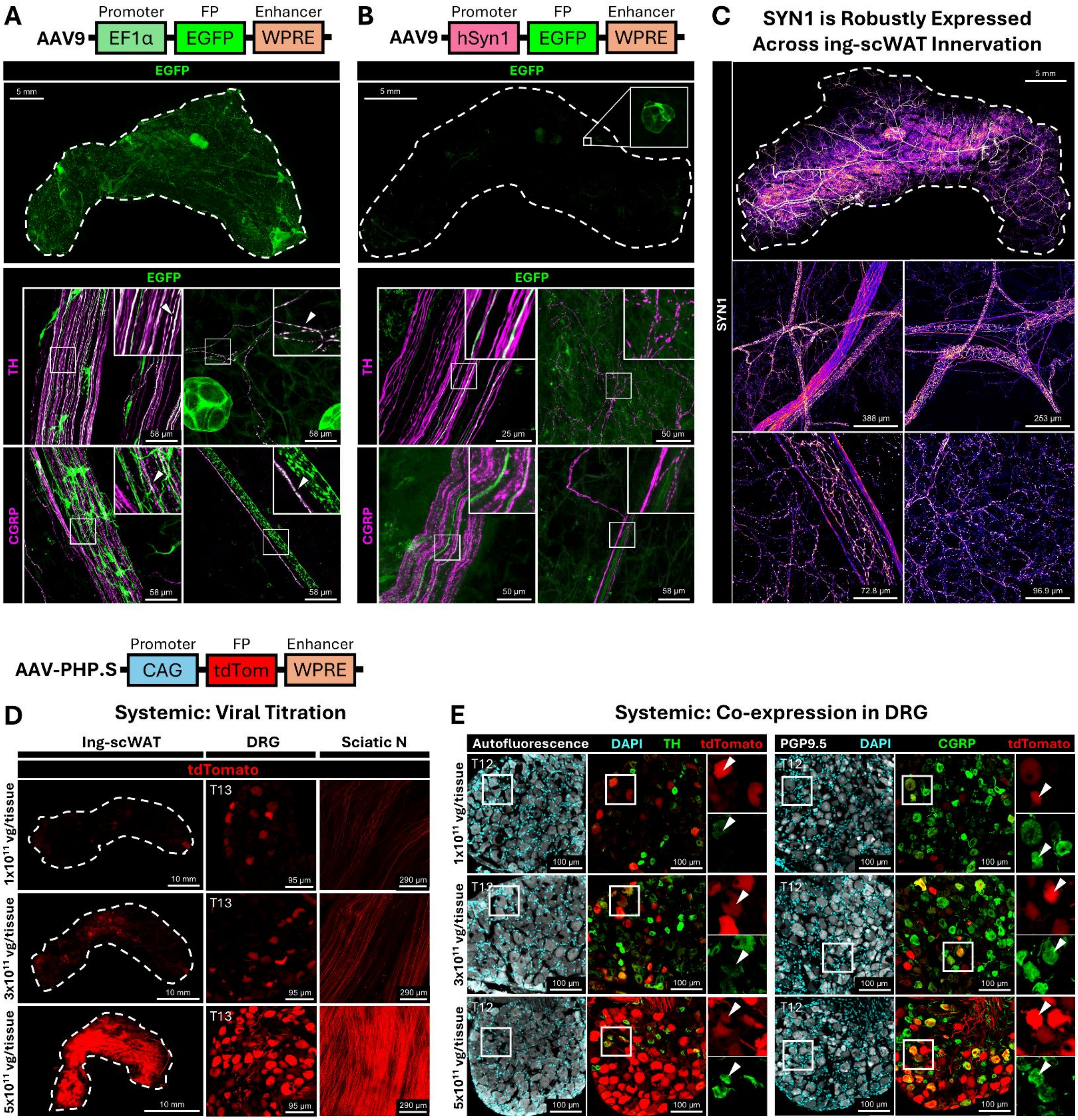

Supplemental Figure S2: Promoter sequences and titration for systemic delivery, related to Figure 1.

- **(A)** AAV9-EF1α-EGFP-WPRE was injected systemically ($1x10^{12}$ vg/mouse, r.o.) into a male C57BL/6J mouse (19-wk-old). Fluorescent transgene expression (EGFP in green) was visualized across the entire ing-scWAT depot, including TH (magenta) and CGRP (magenta) co-labeling (white axons, indicated with arrows).
- **(B)** AAV9-hSyn1-EGFP-WPRE was injected systemically ($1x10^{12}$ vg/mouse, r.o.) into a male C57BL/6J mouse (20-wk-old). Fluorescent transgene expression (EGFP in green) was visualized across the entire ing-scWAT depot, including TH (magenta) and CGRP (magenta) co-labeling.
- **(C)** Ing-scWAT excised from an adult male C57BL/6J mouse (22-wk-old) immunolabeled against SYN1 (LUT; Fire). Representative images of nerve bundles, neurovascular innervation, and parenchymal innervation.
- **(D)** Systemic delivery of AAV-PHP.S-CAG-tdTomato-WPRE to male and female C57BL/6J mice (10-wk-old). Fluorescent transgene expression was compared for $1x10^{11}$, $3x10^{11}$, and $5x10^{11}$ vg/mouse (r.o.). Tissues assessed included intact ing-scWAT, T13 DRG, and sciatic nerve.
- **(E)** Thin-sectioned T12 DRG immunostained against either TH (green) or CGRP (green) to assess the impact of viral titer on co-expression. Autofluorescent / PGP9.5 labeled cell bodies (grey) and DAPI labeled nuclei (cyan). White boxes indicate zoomed regions, and white arrows point to examples of co-labeled cells (yellow).

Supplemental Figure S3: Troubleshooting AAV-PHP.S-FLEX in ing-scWAT, related to Figure 3.

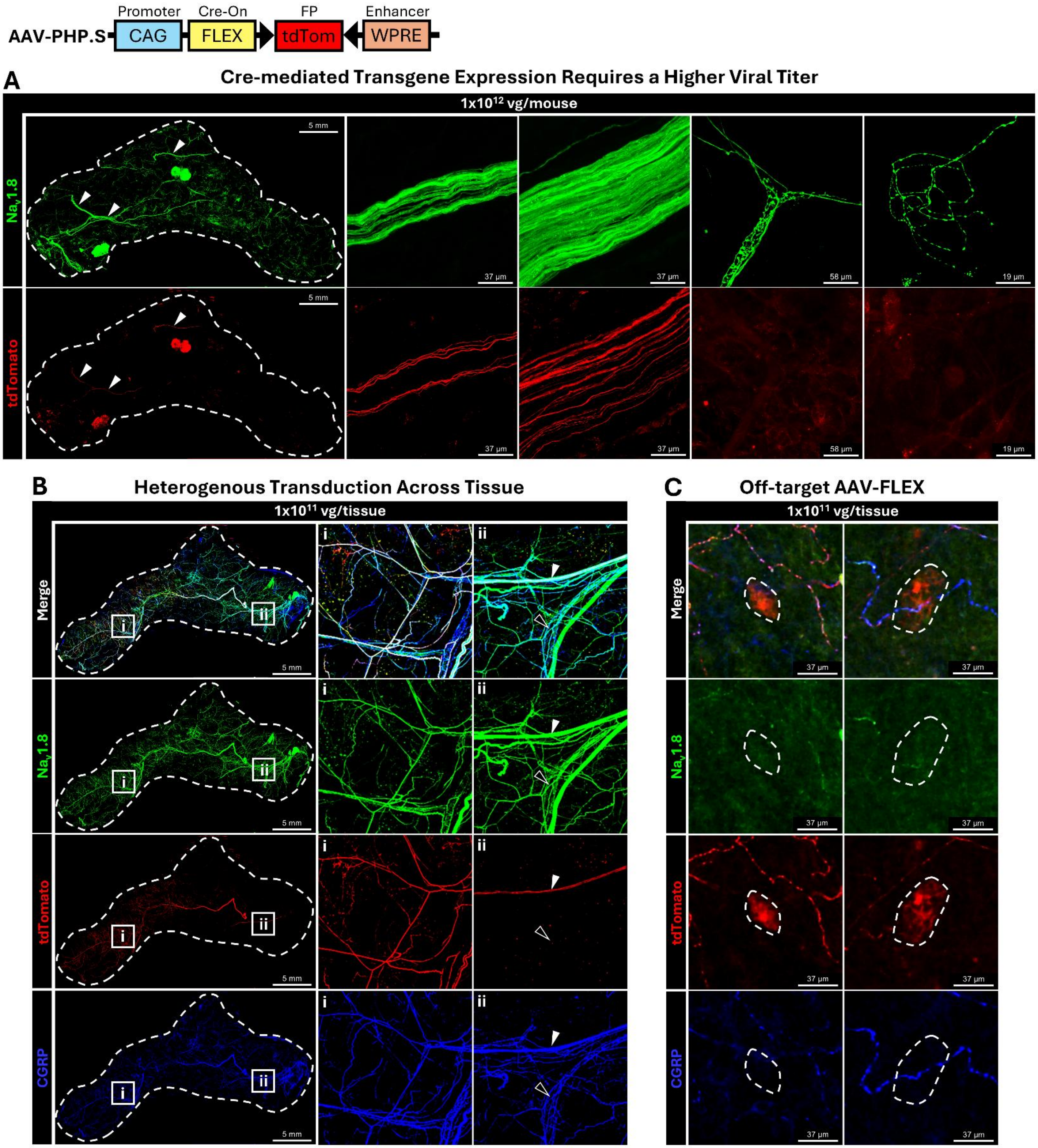

Supplemental Figure S3: Troubleshooting AAV-PHP.S-FLEX in ing-scWAT, related to Figure 4.

- **(A)** AAV-PHP.S-CAG-FLEX-tdTomato-WPRE was injected systemically ($1x10^{12}$ vg/mouse, r.o.) into a male $Na_v1.8$-Cre::ZsGreen1 mouse (33-wk-old). Cre-mediated tdTomato transgene expression was limited to just the largest nerve bundles in ing-scWAT (indicated by white arrows). This indicated that a higher viral concentration was required for Cre-dependent (FLEX/DIO) AAVs in the periphery.
- **(B)** AAV-PHP.S-CAG-FLEX(tdTomato)-WPRE injected into ing-scWAT (5 µL of $1x10^{11}$ vg, i.a.) of a male $Na_v1.8$-Cre::ZsGreen1 mouse (12-wk-old) demonstrated heterogeneous/uneven transgene expression across the ing-scWAT depot, suggesting the need for higher volumes and/or multiple microinjections across the tissue. (i) Close to the injection site, (ii) far from the site; transduced axons (white arrow), non-transduced axons (black arrow).
- **(C)** Off-target Cre-mediated transgene expression was observed in a few adipocytes in ing-scWAT (white dashed line).

Supplemental Figure S4: Brainbow was not as effective as Tetbow in ing-scWAT, related to Figure 6.

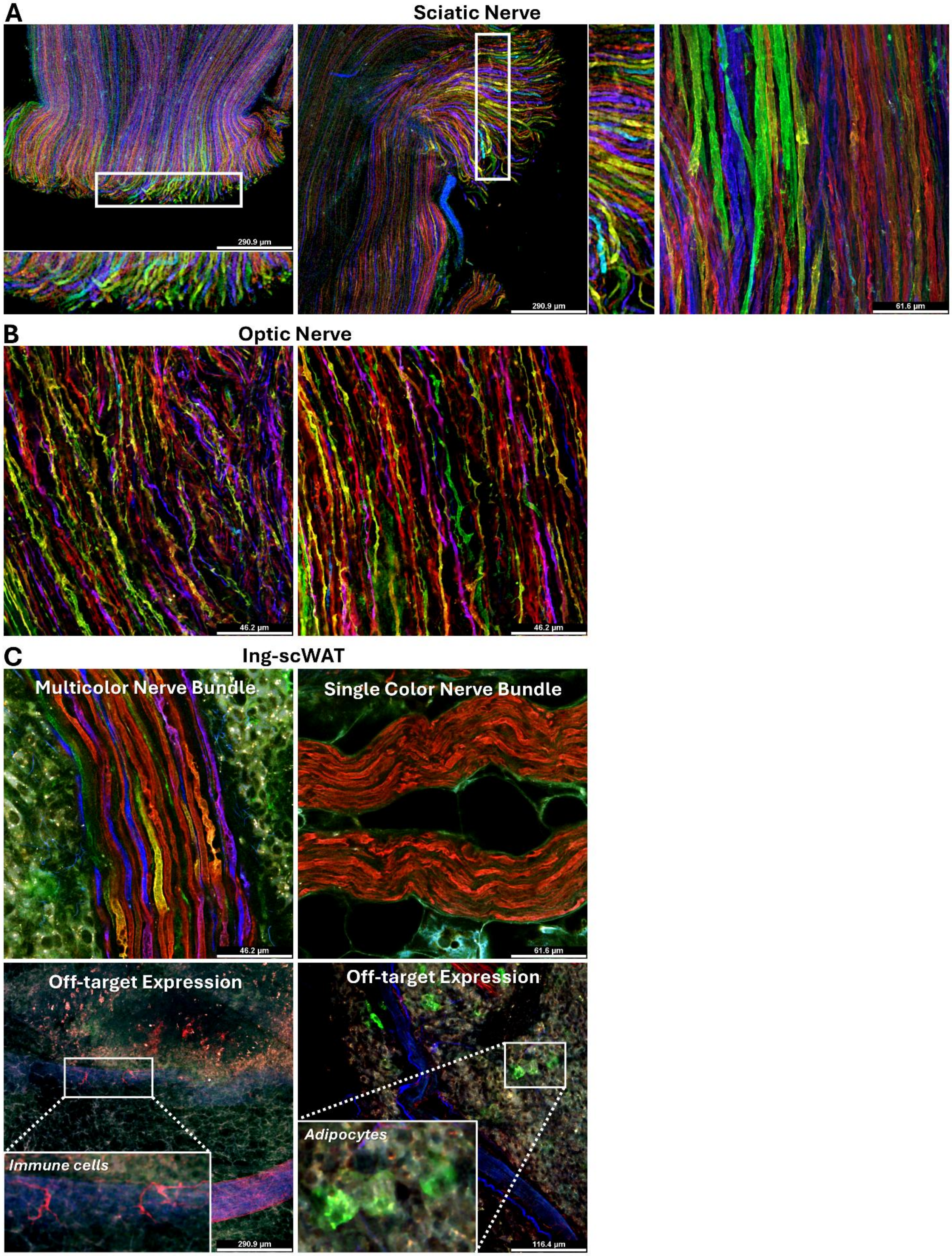

Supplemental Figure S4: Brainbow was not as effective as Tetbow in ing-scWAT, related to Figure 6.

(A) Male CMV-Cre::Brainbow3.2 mice (12-wk-old) used to drive multicolor labeling of adjacent axons in the sciatic nerve.

(B) Multicolor labeling of adjacent axons in the optic nerve.

(C) A single ing-scWAT nerve bundle presented with multicolor labeling. The rest were labeled by single colors. Additionally, off-target fluorescent protein expression was observed in immune cells and adipocytes.

Supplemental Figure S5: Design of AAV-DREADD plasmid, related to Figure 7.

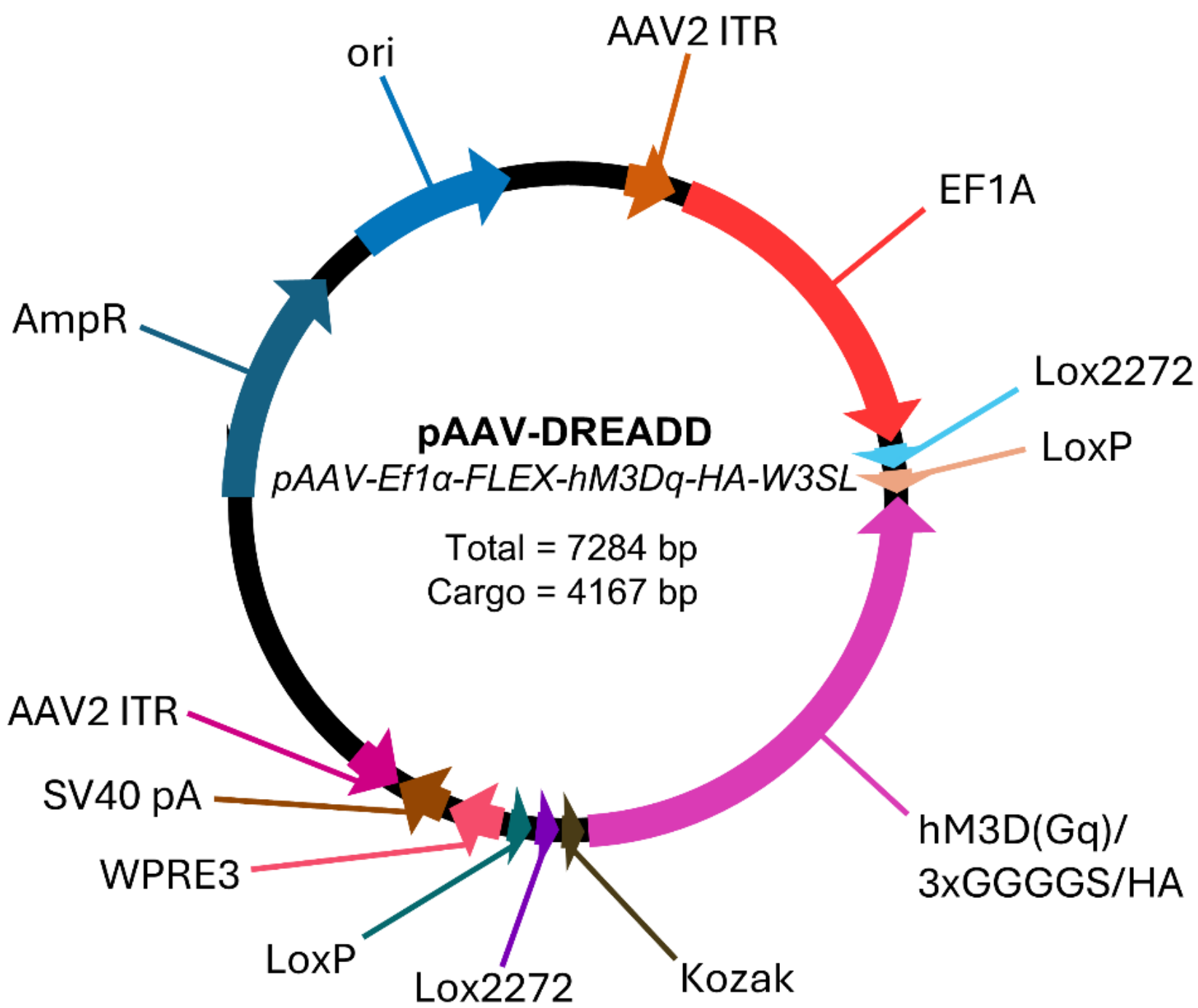

Name	Position	Size (bp)	Description
**AAV2 ITR**	237-377	141	Inverted terminal repeats required for replication
**EF1A**	434-1612	1179	Human eukaryotic translation elongation factor 1 α1; strong ubiquitous promoter
**Lox2272**	1637-1670	34	Recognition site of Cre recombinase
**LoxP**	1703-1736	34	Recognition site of Cre recombinase
**hM3D(Gq)/ 3xGGGGS/ HA**	Complement 1749-3614	1866	Excitatory DREADD receptor with fused hemagglutinin (HA) tag
**Kozak**	Complement 3615-3620	6	Translation start site
**Lox2272**	Complement 3627-3660	34	Recognition site of Cre recombinase
**LoxP**	Complement 3693-3726	34	Recognition site of Cre recombinase
**WPRE3**	3751-4001	251	Truncated form of WPRE enhancer which contains two of the three regulatory elements
**SV40 pA**	4002-4182	181	Late polyadenylation signal
**AAV2 ITR**	Complement 4263-4403	141	Inverted terminal repeats required for replication
**AmpR**	5484-6344	861	Allows for ampicillin resistance
**ori**	6515-7103	589	Facilitates plasmid replication

Supplemental Figure S5: Design of AAV-DREADD plasmid, related to Figure 6.

AAV-DREADD plasmid design developed with VectorBuilder for this manuscript.

Supplemental Figure S6: AAV-DREADD and intravital calcium imaging, related to Figure 7.

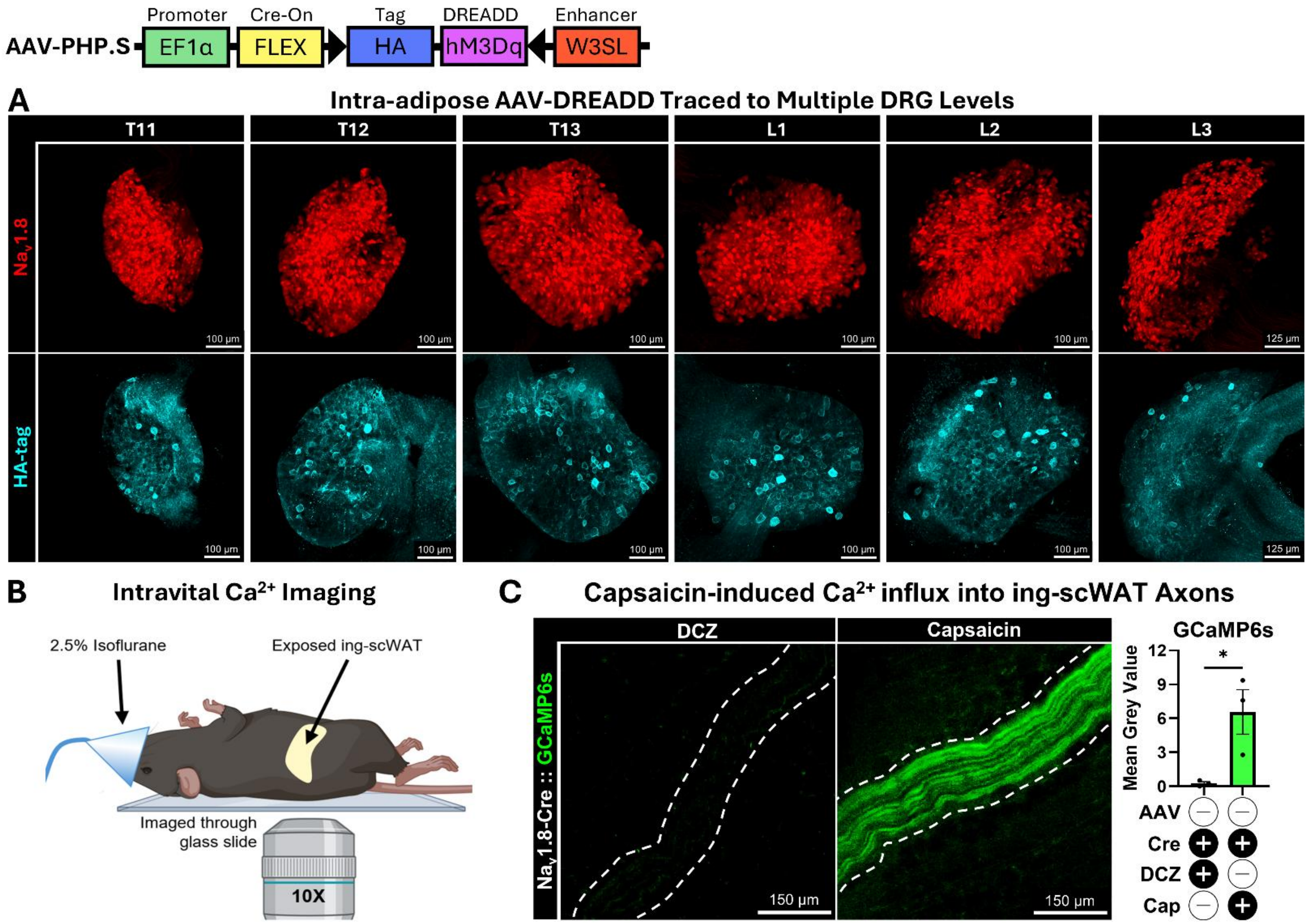

Supplemental Figure S6: Intravital calcium imaging of AAV-DREADD transduced sensory axons in ing-scWAT, related to Figure 6.

(A) Male and female $Na_v1.8$-Cre::tdTomato mouse (n = 6, 9-15-wk-old) received intra-adipose injection of AAV-DREADD into ing-scWAT. Intact DRGs were optically cleared and immunostained against the HA-tag. AAV-DREADD transduction was traced to neurons at multiple levels of DRG that innervate ing-scWAT (T11-L3).

(B) Schematic showing mouse positioning for intravital imaging.

(C) Calcium imaging of the ing-scWAT depot of male and female $Na_v1.8$-Cre::GCaMP6s mice (that did not receive AAV-DREADD)(n = 3). The ing-scWAT depot was exposed and bathed in 100 µL of a denervating capsaicin solution (20 µg/µL). GCaMP6s fluorescence intensity was compared against that of mice that received DCZ alone. *$p < 0.05$, by Student's t-test. Error bars are ± SEM.

Supplemental Table ST1: Overview of viral vectors, related to Figure 1-7.

Capsid	Promoter	Cargo	Source	Injection	Titer
AAV1	CAG	tdTomato	Addgene; 59462-AAV1	RO	$1x10^{12}$ vg/mouse
AAV5	CAG	tdTomato	Addgene; 59462-AAV5	RO	$1x10^{12}$ vg/mouse
AAV9	CAG	tdTomato	Addgene; 59462-AAV9	RO	$1x10^{12}$ vg/mouse
AAV9	CAG	tdTomato	Addgene; 59462-AAV9	IA	$1x10^{12}$ vg/tissue
AAV9	hSyn	EGFP	Addgene; 50465-AAV9	RO	$1x10^{12}$ vg/mouse
AAV9	EF1α	EGFP	Addgene; 105547-AAV9	RO	$1x10^{12}$ vg/mouse
AAVrg	CAG	tdTomato	Addgene; 59462-AAVrg	RO	$1x10^{12}$ vg/mouse
AAV-PHP.S	CAG	tdTomato	Addgene; 59462-PHP.S	RO	$1x10^{12}$ vg/mouse<br>$5x10^{11}$ vg/mouse<br>$3x10^{11}$ vg/mouse<br>$1x10^{11}$ vg/mouse
AAV-PHP.S	CAG	tdTomato	Addgene; 59462-PHP.S	IA	$1x10^{12}$ vg/tissue<br>$5x10^{11}$ vg/tissue<br>$3x10^{11}$ vg/tissue<br>$1x10^{11}$ vg/tissue
AAV-PHP.S	CAG	FLEX-tdTomato	Addgene; 28306-PHP.S	RO	$3x10^{12}$ vg/mouse<br>$1x10^{12}$ vg/mouse
AAV-PHP.S	EF1α	FLEX-HA-hM3Dq	Vector Builder for plasmid design, CPP Vector Core for viral packaging	IA	$3x10^{11}$ vg/tissue
AAV-PHP.S	CAG	DIO-tTA	CPP Vector Core	IA	$1x10^{10}$ vg/tissue<br>$1x10^{11}$ vg/tissue<br>$3x10^{11}$ vg/tissue
AAV-PHP.S	TRE	DIO-mTurqoise2	CPP Vector Core	IA	$3x10^{11}$ vg/tissue
AAV-PHP.S	TRE	DIO-mNeonGreen1	CPP Vector Core	IA	$3x10^{11}$ vg/tissue
AAV-PHP.S	TRE	DIO-tdTomato	CPP Vector Core	IA	$3x10^{11}$ vg/tissue
AAV-MaCPNS1	CAG	EGFP	CPP Vector Core	RO	$1x10^{12}$ vg/mouse
AAV-MaCPNS2	CAG	EGFP	CPP Vector Core	RO	$1x10^{12}$ vg/mouse

CPP Vector Core = California Pamona Polytechnic University NIH BRAIN Initiative Armamentarium Vector Core

RO = Systemic delivery by retro-orbital injection
IA = Intra-adipose microinjection into ing-scWAT

Supplemental Table ST1: Overview of viral vectors, related to Figures 1-7

Comprehensive list of AAV vectors with corresponding promoters, cargo, routes of administration, and viral titers.

Supplemental Table ST2: Antibody list, related to Figures 1-7.

Tissue	Antibody	RRID	Vendor	Catalog #	Concentration
Whole Mount Adipose	TH	AB_390204	EMD Millipore	ab152	1:500
	CGRP	AB_2798662	Cell Signaling	14959	1:500
	SYN1	AB_2616578	Cell Signaling	5297	1:250
	HA-tag (AF594)	AB_2924897	Cell Signaling	45346	1:200
	GFP (AF488)	AB_221477	ThermoFisher	A21311	1:200
	Goat anti-Rabbit (AF594)	AB_2762824	ThermoFisher	A32740	1:1000
	Goat anti-Rabbit (AF647)	AB_2633282	ThermoFisher	A32733	1:500
Soleus NMJs	2H3	AB_531793	DSHB	2H3	1:500
	SV2	AB_2315387	DSHB	SV2	1:250
	Goat anti-mouse (AF647)	AB_2535804	ThermoFisher	A21121	1:500
DRGs	PGP9.5 (AF488)	NA	Abcam	ab302664	1:200
	PGP9.5 (AF647)	NA	Abcam	ab196173	1:200
	TH	AB_390204	EMD Millipore	ab152	1:500
	CGRP	AB_2798662	Cell Signaling	14959	1:500
	HA-tag	AB_1549585	Cell Signaling	3724	1:200
	HA-tag (AF594)	AB_2924897	Cell Signaling	45346	1:200
	Goat anti-Rabbit (AF594)	AB_2762824	ThermoFisher	A32740	1:1000
	Goat anti-Rabbit (AF647)	AB_2633282	ThermoFisher	A32733	1:500

Supplemental Table ST2: Antibody list, related to Figures 1-7.

List of primary and secondary antibodies used throughout the manuscript.
